# Vacuolar zinc transporter TaMTP1 safeguards wheat fertility and regulates grain zinc allocation via stem‐mediated sequestration

**DOI:** 10.1111/tpj.70870

**Published:** 2026-04-16

**Authors:** Yongfang Wan, Deyong Zhao, Rui Tang, Suzanne J Clark, Anjana Magaji‐Umashankar, Petros P Sigalas, Malcolm J Hawkesford

**Affiliations:** ^1^ Sustainable Soils and Crops Rothamsted Research Harpenden Hertfordshire AL5 2JQ UK; ^2^ College of Biological and Pharmaceutical Engineering Shandong University of Aeronautics 391 Huanghe 5th Road Binzhou Shandong 256600 China; ^3^ Triticeae Research Institute, Sichuan Agricultural University No. 211 Huimin Road Chengdu Sichuan 611130 China; ^4^ Intelligent Data Ecosystems Rothamsted Research Harpenden Hertfordshire AL5 2JQ UK; ^5^ Bioimaging, Plant Sciences and the Bioeconomy Rothamsted Research Harpenden Hertfordshire AL5 2JQ UK

**Keywords:** *TaMTP1*, vacuolar Zn transporter, wheat fertility, Zn homeostasis, grain Zn content

## Abstract

Metal tolerance protein 1 (MTP1) is essential for metal homeostasis and detoxification, but its roles in crop fertility and reproductive organ development are poorly understood. Here, we demonstrate that wheat TaMTP1 safeguards reproductive success and grain Zn allocation by mediating vacuolar Zn sequestration in stems. Heterologous expression in yeast and subcellular localization confirmed that TaMTP1 functions as a tonoplast Zn transporter. In wheat, the *TaMTP1* gene was highly expressed in developing spikes and stems across all growth stages. Triple knockout mutants (aabbdd) from a TILLING population exhibited sterility, stunted growth, and abnormal pollen and stigma development, particularly under high Zn supply. ICP‐OES analysis revealed Zn hyperaccumulation in reproductive tissues due to impaired vacuolar sequestration in stems, leading to excessive Zn translocation to spikes. Fluorescence imaging showed Zn overaccumulation in pollen, and depletion in the phloem of stem vascular bundles. Fertility and normal growth were restored under low Zn condition, confirming that reproductive failure was driven by Zn toxicity rather than deficiency. Single functional *TaMTP1* alleles were sufficient to maintain fertility, whereas partial mutants (Aa/AAbbdd and aaBbdd) accumulated elevated Zn in grain under excess Zn supply compared with null. Together, our findings uncover a stem‐based vacuolar Zn buffering mechanism that prevents reproductive Zn toxicity, establishing *TaMTP1* as a key determinant of fertility and grain Zn partitioning in wheat, with potential for biofortification and Zn resilience breeding.

## Significance Statement

The TaMTP1 protein regulates Zn delivery to reproductive organs and grains by mediating stem vacuolar sequestration, a critical mechanism of reproductive Zn homeostasis safeguarding crop productivity under fluctuating Zn supply and providing a genetic entry point for Zn‐biofortified wheat with enhanced nutritional quality.

## INTRODUCTION

Zinc (Zn) is an essential micronutrient required for multiple physiological processes in plants, including enzyme activation, photosynthesis, and hormone biosynthesis (Hacisalihoglu et al., 2003; Wang et al., 2021). Both Zn deficiency and excess can inhibit plant growth, development, and productivity (Kandil et al., 2022; Meng et al., 2023; Pathak et al., 2012; Pathak & Pandey, 2010). To maintain Zn homeostasis, plants have developed a complex regulatory network that tightly controls Zn concentrations at the organ, cellular, and subcellular levels in response to both environmental fluctuations and developmental demands. The vacuole plays a central role in metal ion storage, with Zn sequestration into vacuoles serving as a key mechanism for detoxification and cellular Zn homeostasis (Bashir et al., 2016; Zhao et al., 2022).

Metal Tolerance Protein 1 (MTP1) is recognized as a vacuolar Zn transporter that mitigates Zn toxicity by facilitating vacuolar sequestration. Its function has been demonstrated in several plant species through the rescue of Zn‐hypersensitive yeast mutants under excess Zn conditions (Blaudez et al., 2003; Ricachenevsky et al., 2013; Wang et al., 2018). However, functional studies on MTP1 in plants remain largely limited to the model species Arabidopsis and rice. In *Arabidopsis thaliana*, *AtMTP1* is highly expressed in dividing and differentiating cells of the root, shoot, flowers, and seeds. Disruption of *AtMTP1* through RNA interference or T‐DNA insertion increased root hypersensitivity to excess Zn and reduced Zn concentrations in vegetative tissues (Desbrosses‐Fonrouge et al., 2005; Kawachi et al., 2009). In rice, CRISPR‐mediated knockout of *OsMTP1* enhanced Zn translocation from the roots to rice grains by reducing Zn sequestration into vacuoles in the roots (Ning et al., 2023). This finding contrasts with earlier reports on *OsMTP1* knockdown lines generated via RNA interference (RNAi), where Zn concentrations in both grains and vegetative tissues decreased under Zn addition (Yuan et al., 2012). These inconsistencies may be due to methodological differences or Zn treatment conditions. Additionally, overexpression of *PtdMTP1* and *OsMTP1* in Arabidopsis increased Zn tolerance (Blaudez et al., 2003; Menguer et al., 2013). In *Thlaspi goesingense*, *TgMTP1* overexpression and grafting experiments highlighted its role in inducing systemic Zn deficiency responses and upregulating other Zn transporters (Gustin et al., 2009).

Zn homeostasis is essential for plant fertility and productivity, particularly during the reproductive stage. Both Zn deficiency and toxicity can impair pollen development, reducing pollen viability and ultimately affecting seed set and crop yield (Kandil et al., 2022; Mohsenzadeh et al., 2011; Pandey et al., 2006; Sharma et al., 1979, 1990). Several Zn transporters have been identified as key regulators of Zn homeostasis in reproductive organs. In Arabidopsis and tobacco, double mutants of *athma2/4* or *nthmaα/β* exhibited impaired pollen development and germination, leading to sterility or reduced seed production due to insufficient Zn translocation from roots to reproductive organs (Hermand et al., 2014; Hussain et al., 2004). In rice, OsMT2b/2c metallothioneins regulate Zn allocation in the panicle, ensuring proper anther development and seed set by modulating Zn transport from the upper node (Lei et al., 2021).

Despite the well‐established roles of MTP1 transporters in metal homeostasis, the specific contribution of TaMTP1 to wheat fertility and grain Zn allocation remains unexplored. Understanding the molecular mechanisms of Zn transport in wheat is essential for developing Zn‐efficient and Zn‐biofortified crop varieties, particularly in regions with fluctuating soil Zn levels. Here, we investigate the role of TaMTP1 in Zn homeostasis, reproductive development, and grain Zn partitioning. By analyzing *TaMTP1* expression patterns, mutant phenotypes, and Zn distribution, we reveal how TaMTP1 maintains reproductive success and influences grain Zn accumulation by regulating Zn translocation from stem to reproductive organs. These insights highlight TaMTP1 as a promising target for breeding strategies aimed at enhancing both fertility and Zn biofortification in wheat.

## RESULTS

### Heterologous expression of *TaMTP1* in yeast, subcellular localization, and expression patterns in wheat

**Figure 1:**
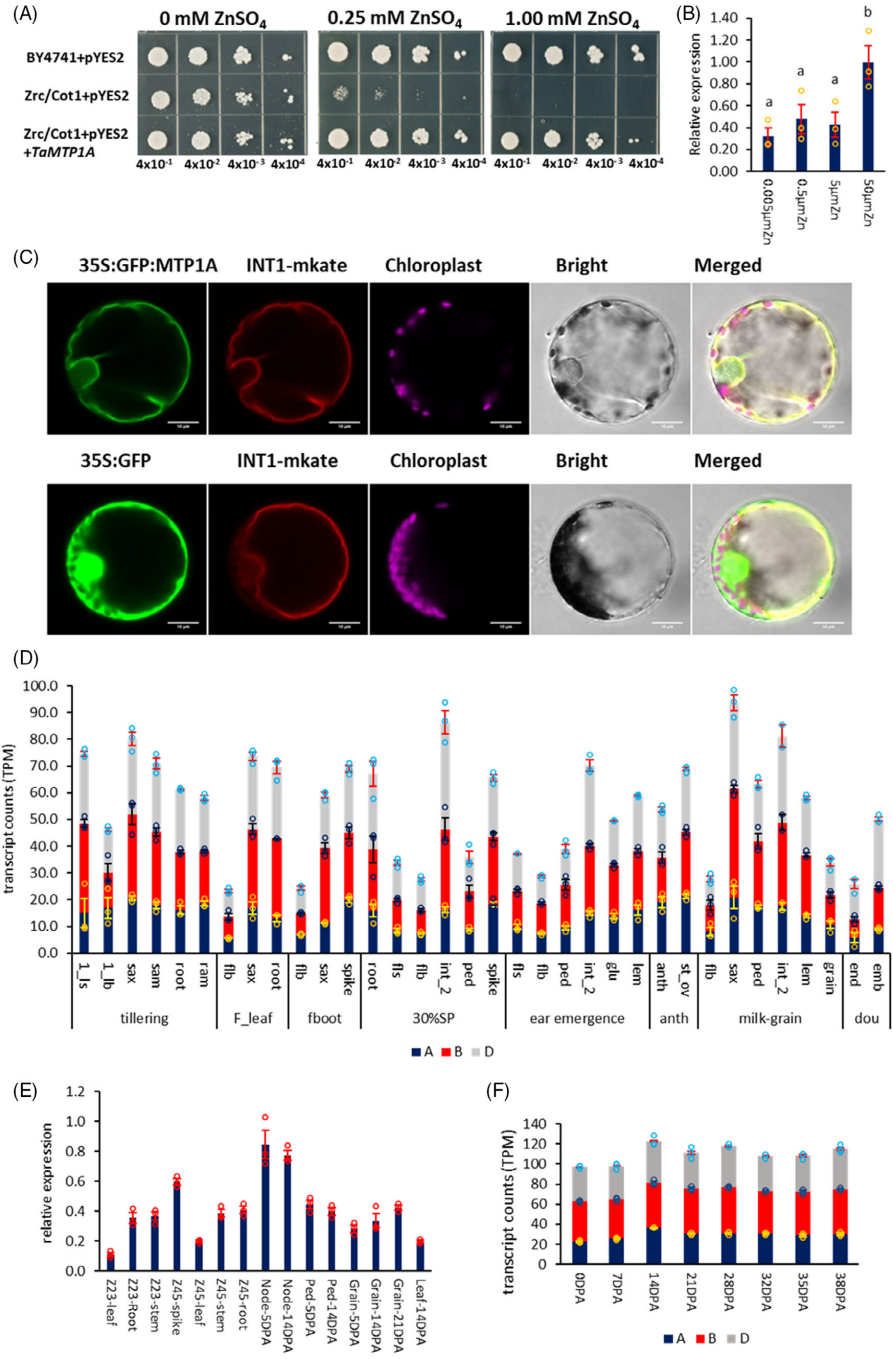
Function, subcellular localization and expression analysis of *TaMTP1*. (A) Wild‐type yeast strain BY4741 was transformed with empty vector pYES2; mutant yeast zrc1/cot1 was transformed with pYES2 or with *TaMTP1*A + pYES2. Serial dilutions of yeast cells at OD_600_ = 4 × 10^−1^, 4 × 10^−2^, 4 × 10^−3^, and 4 × 10^−4^ were sequentially dropped onto and grown on the SC galactose without uracil drop‐out medium with 0, 0.25, and 1 mM ZnSO_4_. (B) *TaMTP1* relative expression by real‐time RT‐PCR in shoot of wheat variety (Cadenza) 2‐week‐old seedling in hydroponic solution with 0.005 μM, 0.5 μM, 5 μM, and 50 μM ZnSO_4_. Data are means ± SE (standard error) from three biological replicates (*n* = 3). Different letters indicate significant differences as determined by one‐way ANOVA followed by an LSD (least significant difference) test for mean comparisons (*P* < 0.05). (C) subcellular localization of TaMTP1A in wheat protoplasts. (D) *TaMTP1* expression in different tissues and growth stages from public RNA‐seq data (*n* = 3) of variety (Azhurnaya) (Ramírez‐González et al., 2018). 1_ls (1st leaf sheath), 1_lb (1st leaf blade), sax (shoot axis), sam (shoot apical meristem), ram (root apical meristem), flb (flag leaf blade), fls (flag leaf sheath), int_2 (internode below the flag leaf node), ped (peduncle), anth (anther), st_ov (stigma and ovary), glu (glumes), lem (lemma); tillering (tillering stage), F‐leaf (flag leaf stage), fboot (full booting stage), 30% SP (30% spike out of sheath), anth (anthesis stage), milk‐grain (grain‐filling milk stage), end (endosperm), emb (embryo), TPM (transcripts per million). A: *TaMTP1A*, B: *TaMTP1B*, D: *TaMTP1D*. (E) *TaMTP1* expression (*n* = 3) in different organs of cultivar Hereward by qPCR at Zadoks 23 (2–3 tillers stage), Zadoks 45 (booting stage) and grain developing stage. Ped (peduncle), DPA (days post‐anthesis). (F) *TaMTP1* expression (*n* = 3) from node 1 RNA‐seq data during anthesis (0 DPA) and grain developing stages (7, 14, 21, 28, 32, 35, and 38 DPA) of cultivar Paragon.

To determine whether TaMTP1 functions as a vacuolar membrane Zn transporter in wheat, *TaMTP1A* was expressed in the yeast (*Saccharomyces cerevisiae*) *zrc1/cot1* double mutant, which lacks the ability to sequester Zn into vacuoles and shows Zn sensitivity. *TaMTP1A* was selected as a representative homeolog for functional assays because *TaMTP1A*, *TaMTP1B*, and *TaMTP1D* are homeologs located at corresponding loci on the 1A, 1B, and 1D subgenomes and share 95–97% identical protein sequences and fully conserved transmembrane domains characteristic of MTP‐type Zn transporters (Figure S1b). On Zn‐free medium, both the *zrc1/cot1* mutant and the mutant expressing *TaMTP1A* grew similarly to the wild‐type (Figure 1A). However, under Zn supplementation (0.25 mM and 1 mM), the *zrc/cot1* mutant failed to grow, whereas the mutant expressing *TaMTP1A* restored growth comparable to that of wild‐type yeast. These results demonstrate that TaMTP1 rescues the Zn‐sensitive phenotype of the yeast mutant by facilitating Zn sequestration into vacuoles, confirming its role as a functional vacuolar Zn transporter in yeast.

To assess whether *TaMTP1* expression responds to high Zn levels, we analyzed expression in 2‐week‐old wheat seedlings grown hydroponically under Zn supplementation (0.005, 0.5, 5, and 50 μM ZnSO_4_) by qPCR. *TaMTP1* was strongly induced in shoots under excess Zn (50 μM), but not under low or moderate Zn supply (0.005–5 μM) (Figure 1B). This pattern suggests a role in detoxification by sequestering excess Zn into vacuoles.

To further verify that TaMTP1 functions as a vacuolar membrane Zn transporter, subcellular localization was examined by transient expression of *TaMTP1A* fused to GFP (35S:GFP–*TaMTP1A*) in wheat protoplasts. The GFP fluorescence signals co‐localized with the vacuolar membrane marker INT1 (35S:GFP–INT1‐mKate) (Figure 1C), confirming that *TaMTP1 i*s targeted to the tonoplast and acts as a vacuolar membrane Zn transporter.

Analysis of the public gene expression atlas from wheat cv Azhurnaya (Ramírez‐González et al., 2018) across multiple growth stages (Figure 1D, Figure S1a) showed that *TaMTP1* is broadly expressed, but with distinct spatial and temporal patterns. At the seedling stage, *TaMTP1* is highly expressed in the meristems of shoots and roots. During the transition from vegetative to grain‐filling stages, expression is predominantly observed in the stem axis (internodes and nodes). During spike development and pre‐anthesis stages, *TaMTP1* is strongly expressed in reproductive tissues, including spikes, anthers and carpels (stigma and ovary). Consistent with this, our qPCR and node‐specific RNA‐seq analyses confirmed that *TaMTP1* is highly expressed in node 1 during anthesis and grain filling (Figure 1E,F), a key site for Zn remobilization to developing grains. The three homeologs contribute unequally to total expression, with *TaMTP1B* and *TaMTP1D* expressed at higher levels than *TaMTP1A*. Together, these spatiotemporal patterns indicate that *TaMTP1* supports Zn homeostasis during reproductive development by regulating Zn sequestration and redistribution within the stem–spike transport pathway.

### Generation of triple knockout mutants

**Figure 2:**
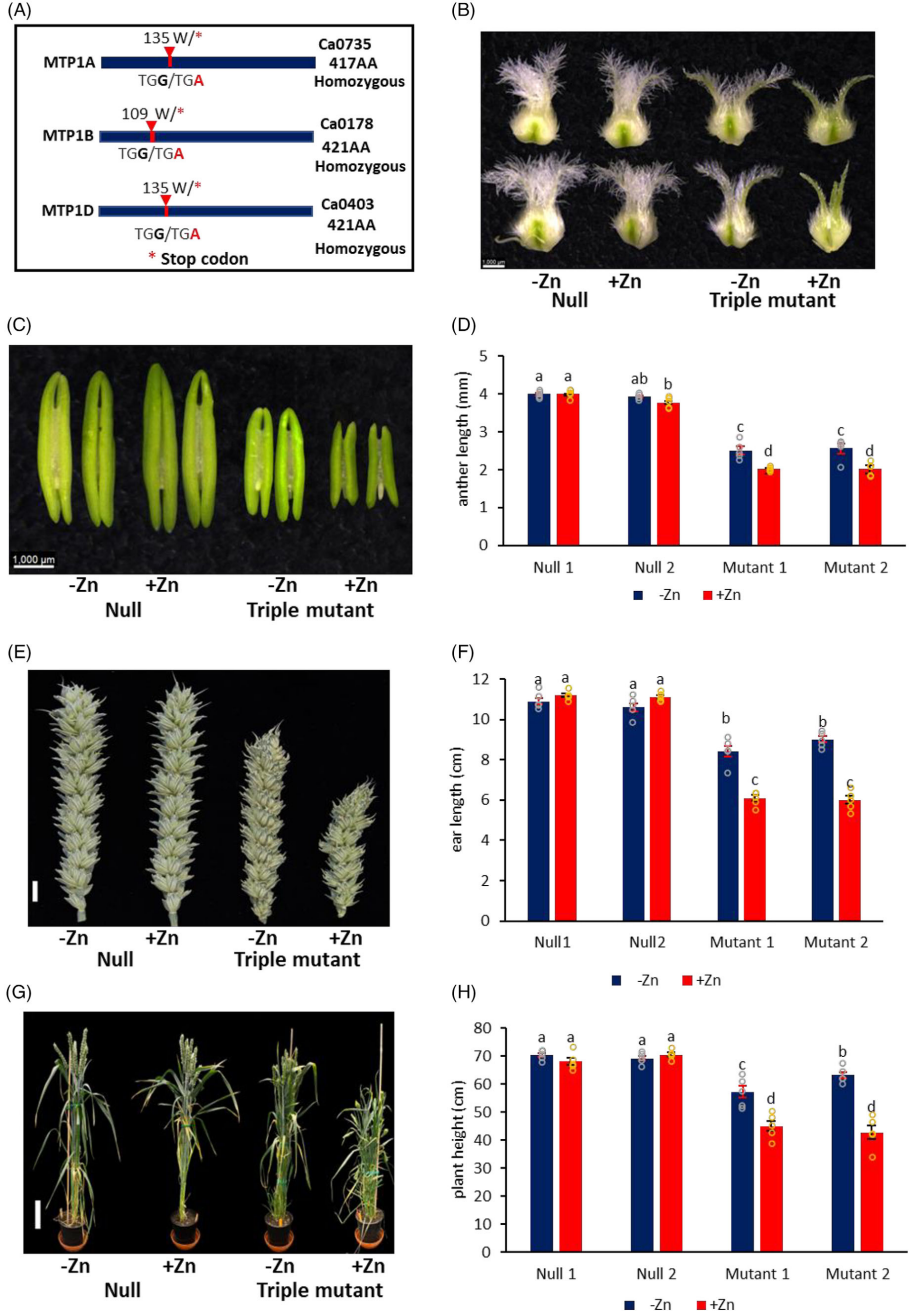
Generation and phenotypes of triple mutants grown in compost without ZnSO_4_·7H_2_O (−Zn) and with 200 mg ZnSO_4_·7H_2_O (+Zn) application per pot. (A) Schematic representation of three homeologs of *TaMTP1A, 1B*, and *1D* and their mutation lines (Ca0735, Ca0178, and Ca0403) from the EMS TILLING population. AA: amino acid. (B) Carpel morphology. (C) Anther morphology. (D) Anther length. (E) Ear morphology. (F) Ear length. (G) Plant morphology. (H) Plant height. Data are means ± SE from five biological replicates (*n* = 5). Different letters indicate significant differences as determined by two‐way multistratum ANOVA followed by an LSD test for mean comparisons (*P* < 0.05) in (D, F, H). Triple mutant 1 (10 individual plants) and triple mutant 2 (10 individual plants) were derived from aabbDd and aaBbdd, respectively. Scale bars represent 1000 μm in (B) and (C), 1 cm in (E), and 10 cm in (G).

To investigate TaMTP1 function in wheat, three in‐frame stop‐gained mutation lines (Ca0735, Ca0178, and Ca0403) on chromosomes A (*TaMTP1A*, TraesCS1A02G071800), B (*TaMTP1B*, TraesCS1B02G090400), and D (*TaMTP1D*, TraesCS1D02G074400) were selected from the EMS hexaploid wheat variety *Cadenza* TILLING (Targeting Induced Local Lesions in Genomes) population (Krasileva et al., 2017; Figure 2A, Figure S1b). Triple knockout mutants were generated by stacking the mutations in all three homeologs and backcrossing with wild‐type *Cadenza to* the BC_3_F_3_ generation (Figure S2a). These triple mutants produced truncated proteins, which may lack Zn transport function.

### Phenotypes of the triple mutants and sensitivity to Zn conditions

The triple mutants (assessed in BC_2_F_2_, BC_2_F_3_, and BC_3_F_2_ plants) exhibited complete sterility with no seed production. As this severe fertility defect is observed only when all three *TaMTP1* homeologs are simultaneously disrupted, the phenotype reflects the combined and redundant function of *TaMTP1A, TaMTP1B*, and *TaMTP1D* rather than the loss of any single *TaMTP1* homeolog. Phenotypic traits, such as floret size, anther length, plant height, ear length and anthesis timing differed under no Zn (−Zn) and Zn supplemented (+Zn) soil conditions. Therefore, BC_3_F_3_ triple mutant plants derived from seeds of BC_3_F_2_ plants, with genotypes *Aabbdd, aaBbdd*, or *aabbDd* were used to further investigate their Zn sensitivity (Figure 2).

Null plants produced significantly larger anthers (3.8–4.0 mm), with their length unaffected by Zn conditions (Figure 2C,D), and released abundant mature yellow pollen at anthesis (Figure S2b). In contrast, triple mutants developed much smaller anthers, with their length decreasing from 2.5 mm (−Zn) to 2 mm (+Zn) (Figure 2C,D). These anthers failed to dehisce and contained immature pollen (Figure S2b). Additionally, triple mutant stigmas exhibited fewer branches under elevated zinc, and some carpels abnormally formed three stigmas (Figure 2B). All the triple mutants in BC_3_F_3_ generation showed sterility both under −Zn and +Zn conditions.

Zn supplementation also resulted in a significant reduction in plant height (42.8–45.0 cm) and ear length (6.0–6.1 cm) in triple mutants compared with null plants (plant height 68.2–70.6 cm and ear length 10.6–11.2 cm) (Figure 2E–H). While flowering time in triple mutants remained unaffected under −Zn conditions, it was delayed by 2 weeks under +Zn conditions relative to null plants. These results indicate that excess Zn exacerbates Zn sensitivity in triple mutants, impairing both vegetative growth and reproductive organ development.

### Reproductive defects and pollen viability in triple mutants

**Figure 3:**
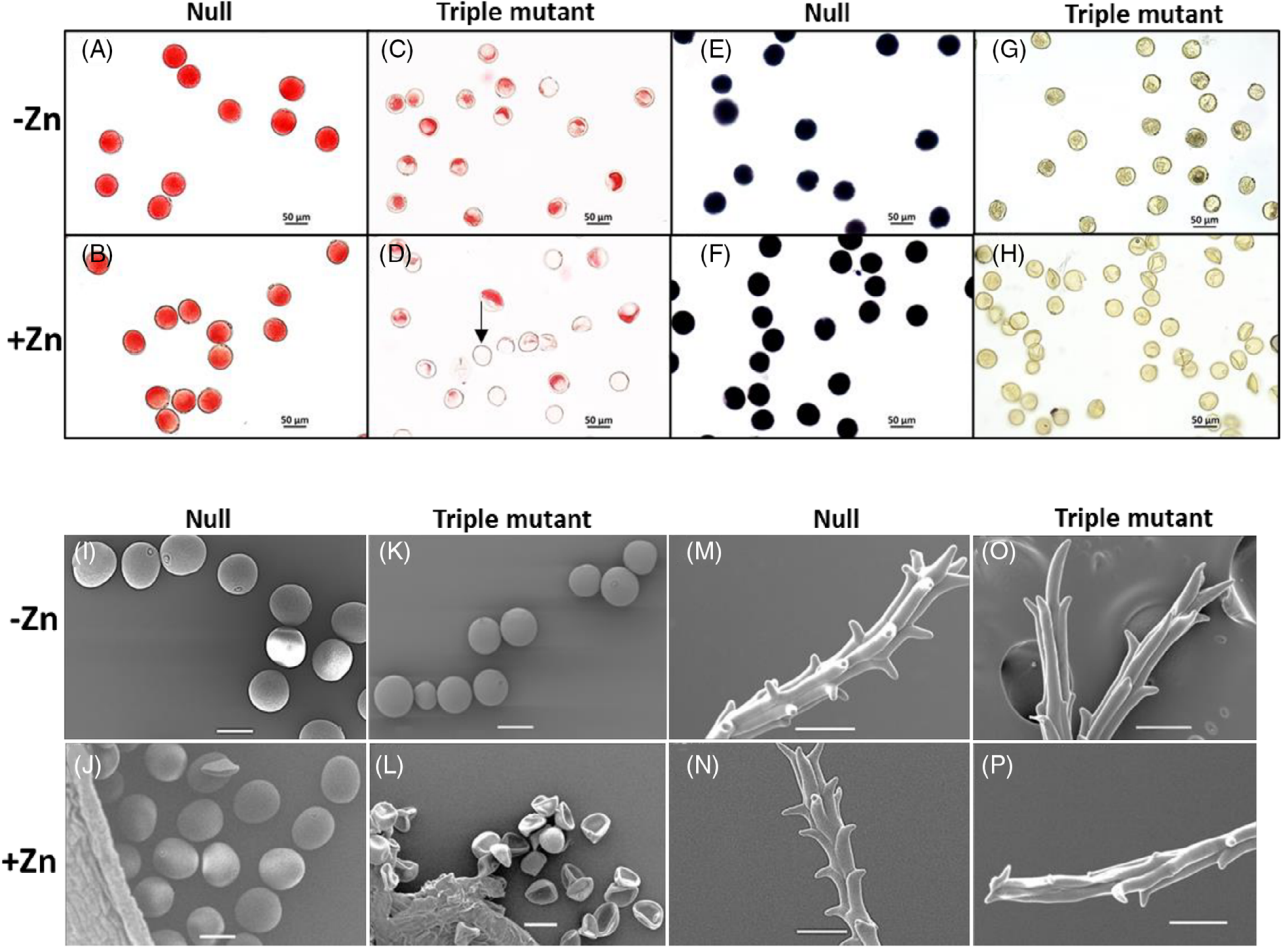
The viability of pollen and scanning morphology of pollen and stigma. Viability assessment of pollen using Alexander staining (A–D) and I_2_‐KI solution staining for starch accumulation in pollen (E–H) in null (A, B, E, F) and triple mutant (C, D, G, H) grown in compost (−Zn) (A, C, E, G) and compost with 200 mg ZnSO_4_·7H_2_0 (+Zn) application per pot (B, D, F, H). Arrow indicates the empty pollen. The morphology of pollen and stigma with scanning microscopy (I–P). Pollen of null (I, J) and triple mutant (K, L), stigma of null (M, N) and triple mutant (O, P) from –Zn (I, K, M, O) and +Zn (J, L, N, P) plants. Scale bars represent 50 μm (A–P).

To further investigate male sterility in the triple mutants, pollen viability was assessed using Alexander staining for cytoplasmic content (red) (Figure 3A–D) and iodine staining for starch accumulation (black) (Figure 3E–H). In null plants, mature pollen was larger, with over 98% viability, showing fully filled cytoplasmic content (red) or starch accumulation (black) under both −Zn and +Zn conditions (Figure 3A,B,E,F, Figure S3a,b). However, pollen from triple mutants was smaller, with partially filled cytoplasmic content and lacking starch (Figure 3C,D,G,H, Figure S3a). The percentage of empty pollen increased from 6.3 to 11.1% (−Zn) to 30–50% (+Zn) with 95–100% of pollen being non‐viable under both Zn conditions (Figure S3b).

*In vitro* germination confirmed that pollen from null plants was viable and successfully germinated, while pollen from triple mutants failed to germinate, indicating complete non‐viability (Figure S3c,d). Scanning electron microscopy revealed that most pollen in triple mutant under +Zn conditions was collapsed due to minimal or absent cytoplasmic content (Figure 3L), in contrast to the null (Figure 3J). Under −Zn conditions, pollen morphology did not differ markedly between triple mutants and null plants (Figure 3I,K). In addition, the stigma branch spikes in triple mutants were poorly developed under both −Zn and +Zn conditions (Figure 3O,P) compared with those of nulls (Figure 3M,N).

To identify whether the sterility in triple mutants resulted from male or female defects, manual fertilization and reciprocal crossing were performed. Crosses of mutant (♀) × null (♂) and null (♀) × mutant (♂) plants in BC_2_F_2_ and BC_2_F_3_ showed no seed set, confirming sterility in both pollen and carpel of the triple mutants.

### Zn overaccumulation and localization in reproduction organs of triple mutants at anthesis

**Figure 4:**
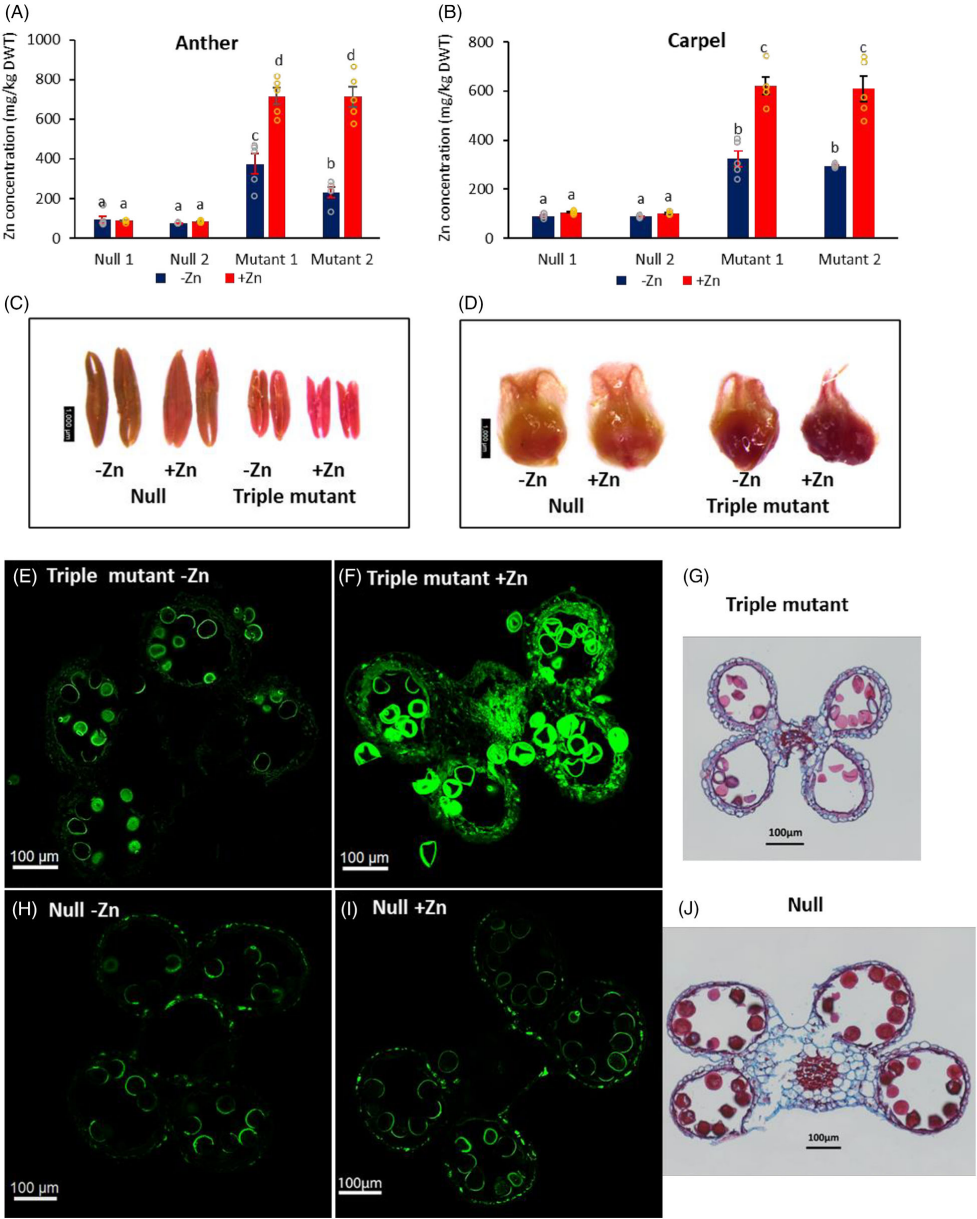
The Zn concentrations and localization in anther and carpel. Zn concentrations in (A) anthers and (B) carpels of nulls and triple mutants grown in compost without ZnSO_4_·7H_2_O (−Zn) and with 200 mg ZnSO_4_·7H_2_O (+Zn) application per pot. Data are means ± SE from five biological replicates (*n* = 5). Different letters indicate significant differences as determined by two‐way multistratum ANOVA followed by an LSD test for mean comparisons (*P* < 0.05). (C) Anther and (D) Carpel Zn staining using dithizone. Zn localization using Zinpyr‐1 and confocal microscope on cryostat cut sections (30 μm thickness) from fresh anthers collected 24–48 h before pollen maturing (E, F, H, I). Fourteen‐micron wax sections of anther were stained using 0.5% Safranin O and 0.1% Astra Blue in 50% ethanol (G and J). Triple mutant sections (E, F, G) and null sections (H, I, J). Scale bars represent 1000 μm in (C) and (D), 100 μm in (E–J).

To explore the association between Zn accumulation in reproductive organs and sterility in the triple mutants, Zn concentrations in anthers and carpels at anthesis were measured under −Zn and +Zn conditions using ICP‐OES. In nulls, Zn concentrations remained low and stable in both organs (77–95 and 89–106 mg/kg dry weight in anthers and carpels respectively) regardless of Zn conditions (Figure 4A,B). By contrast, Zn levels in triple mutant anthers and carpels rose to 232–374 and 297–325 mg/kg respectively, under −Zn, and 713–716 and 609–621 mg/kg respectively, under +Zn conditions. Other metals (Fe and Mn) showed modest increases in triple mutants but were unaffected in nulls (Figure S4).

Dithizone staining revealed a stronger red color in the anthers and carpels of the triple mutants compared with null (Figure 4C,D). Fluorescence Zinpyr‐1 localization of Zn indicated that Zn was primarily concentrated in the pollen of anthers, both in the triple mutants and the nulls under −Zn and +Zn conditions. Additionally, Zn was strongly observed in the vascular bundles of the filament under +Zn conditions in the triple mutant, while in the null anthers, it was prominently observed in the epidermis under both −Zn and +Zn conditions (Figure 4E–I).

These findings suggest that Zn overaccumulation in the reproductive organs leads to toxicity, impairing pollen and ovary stigma development, whereas the nulls maintained low Zn levels, preserving the fertility of the pollen and carpel.

### Altered zinc distribution in triple mutants at anthesis

**Figure 5:**
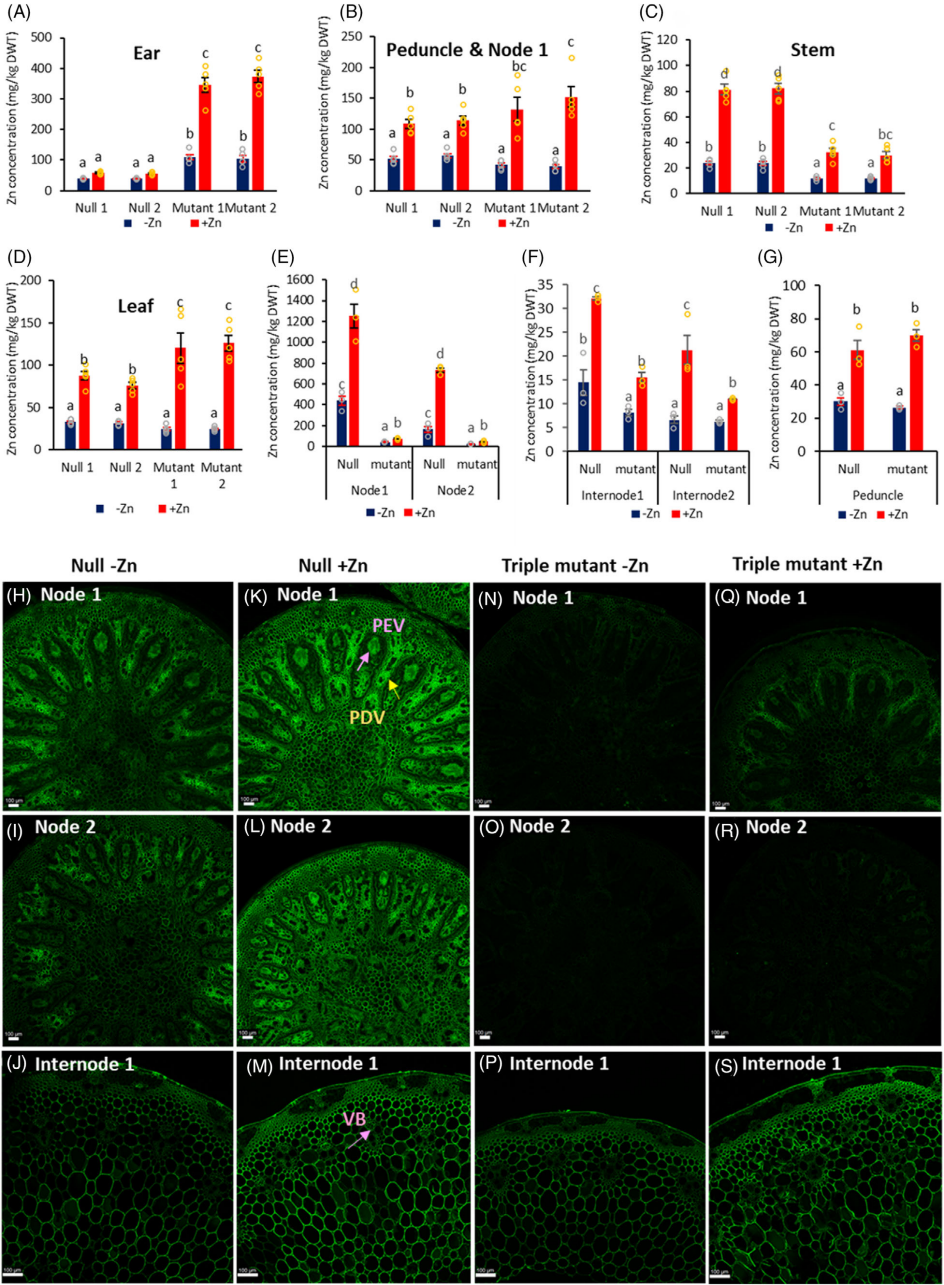
Zn concentrations in different tissues and Zn localization in stem at anthesis grown in compost without ZnSO_4_·7H_2_O (−Zn) and with 200 mg ZnSO_4_·7H_2_O (+Zn) application per pot. Zn concentrations were measured using ICP‐OES in (A) ear, (B) peduncle and flag leaf node 1, (C) stem below node 1, and (D) leaf. Data are means ± SE from five biological replicates (*n* = 5). Different letters indicate significant differences as determined by two‐way multistratum ANOVA followed by an LSD test for mean comparisons (*P* < 0.05). Triple mutant 1 (10 individual plants) and triple mutant 2 (10 individual plants) were derived from (Aabbdd) and (aaBbdd), respectively. (E) node 1 and node 2. (F) internode 1 and internode 2. (G) peduncle. Data are means ± SE from three biological replicates (*n* = 3). Data were analyzed using two‐way single‐stratum ANOVA followed by an LSD test for mean comparisons (*P* < 0.05). Different letters (E–G) indicate significant differences between null and mutant in each tissue (node1, node2, internode 1, internode 2, and peduncle, respectively). Zn localization using Zinpry‐1 and visualized using the Leica Stellaris Falcon confocal at 5 X magnification in flag leaf node 1 (H, K, N, Q), node 2 (I, L, O, R), and at 10X magnification for the internode 1 below flag leaf node 1 (j, m, p, s). PEV: phloem of enlarge vascular bundle; PDV: phloem of diffusion vascular bundle; VB: vascular bundle. Scale bars represent 100 μm (H–S). The negative control for (H–S) is shown in Figure S6.

To investigate the origin of the overaccumulated Zn in the reproductive organs, Zn concentrations were measured using ICP‐OES in different tissues: whole ear (including anthers, carpels, rachis, and glumes), peduncle and node 1, stem (remaining axis) and leaves (including the sheath) at anthesis. In the nulls, Zn concentrations in the ears showed no significant differences between the two Zn conditions (Figure 5A). However, Zn concentrations increased 2‐, 2.5‐, and 3.5‐fold in peduncle, leaf, and stem of the nulls, respectively, under +Zn conditions compared with those under −Zn conditions (Figure 5B–D), indicating that excess Zn was primarily sequestered in these vegetative organs to prevent toxicity and protect reproductive organ development and fertility.

In contrast, Zn concentrations in the ear of the triple mutants increased 2.75‐fold under −Zn conditions and 6.7‐fold under +Zn conditions compared with the nulls. Zn levels in the stem of the triple mutants decreased twofold under −Zn and 2.6‐fold under +Zn conditions compared with the nulls, while they increased 1.5‐fold in leaves under +Zn conditions. These findings suggest that in the triple mutants, Zn was not adequately stored in the stem and was instead translocated to the ear (the sink) at anthesis, leading to Zn overaccumulation.

Other metals, such as Fe and Mn, also significantly increased in the ear of the triple mutants under +Zn condition but did not show a corresponding decrease in the stem like Zn. In addition, Fe and Mn concentrations remained unchanged in the null (Figure S5).

Further dissection of stem tissues revealed markedly reduced Zn concentrations in nodes 1 and 2 of triple mutants compared with nulls. Specifically, node 1 and node 2 in nulls accumulated 10‐fold and 6‐fold more Zn under −Zn, and 16‐fold more under +Zn, respectively (Figure 5E). Although Zn levels in internodes 1 and 2 were lower overall, they were still approximately twofold higher in nulls than in triple mutants under +Zn (Figure 5F). Zn concentrations in the peduncle did not differ between genotypes (Figure 5G).

Zn localization using Zinpyr‐1 fluorescence confirmed these observations (Figure 5H–S). In nulls, strong Zn signals were detected in the phloem of both enlarged vascular bundles and diffuse vascular bundles in nodes (Figure 5H,I,K,L), while triple mutants exhibited very weak or no signals in these tissues under both Zn conditions (Figure 5N,O,Q,R). In the internode, no differences were observed between null and triple mutant under −Zn condition. Zn was clearly localized in ground tissues and vascular bundle cells of nulls (Figure 5J,M), whereas triple mutants showed only weak signals in phloem and xylem parenchyma cells under +Zn conditions, in contrast to the null (Figure 5S), suggesting altered Zn deposition patterns. These data indicate that in wild‐type plants, the phloem of vascular bundles in nodes and parenchyma cells in internodes may function as temporary Zn storage sites, likely mediated by *TaMTP1*‐dependent vacuolar Zn sequestration.

### Restoration of fertility in triple mutant plants under low zinc conditions

The development of reproductive organs in the triple mutants was significantly inhibited by Zn supplementation. We hypothesized that plant growth and fertility could be restored if grown in low Zn soil. To test this, the triple mutants were initially grown in low fertility sandy soil containing 20 mg/kg Zn, which is half the Zn content of compost soil (40 mg/kg dry weight) used for plant growth. Under these conditions, the triple mutants reached the same height as the nulls (Figure S7a), and their carpel morphology appeared normal (Figure S7b). Cross‐pollination of mutant ovules with normal wild‐type (WT) Cadenza pollen produced six seeds from 60 pollinated florets (Figure S7c). Although the six fertilized seeds developed normally during the first 10 days, grain filling was stopped, while null grains developed successfully, indicating the failure of grain filling in the triple mutant did not result from insufficient nutrients from the soil. The pollen in triple mutants remained incompletely filled with cytoplasm (Figure S7d), and most of the pollen lacked starch, with minimal starch accumulation (Figure S7e). No self‐pollinated seeds were produced in the triple mutant plants, indicating that although the carpel had restored partial fertility, the pollen still remained sterile.

**Figure 6:**
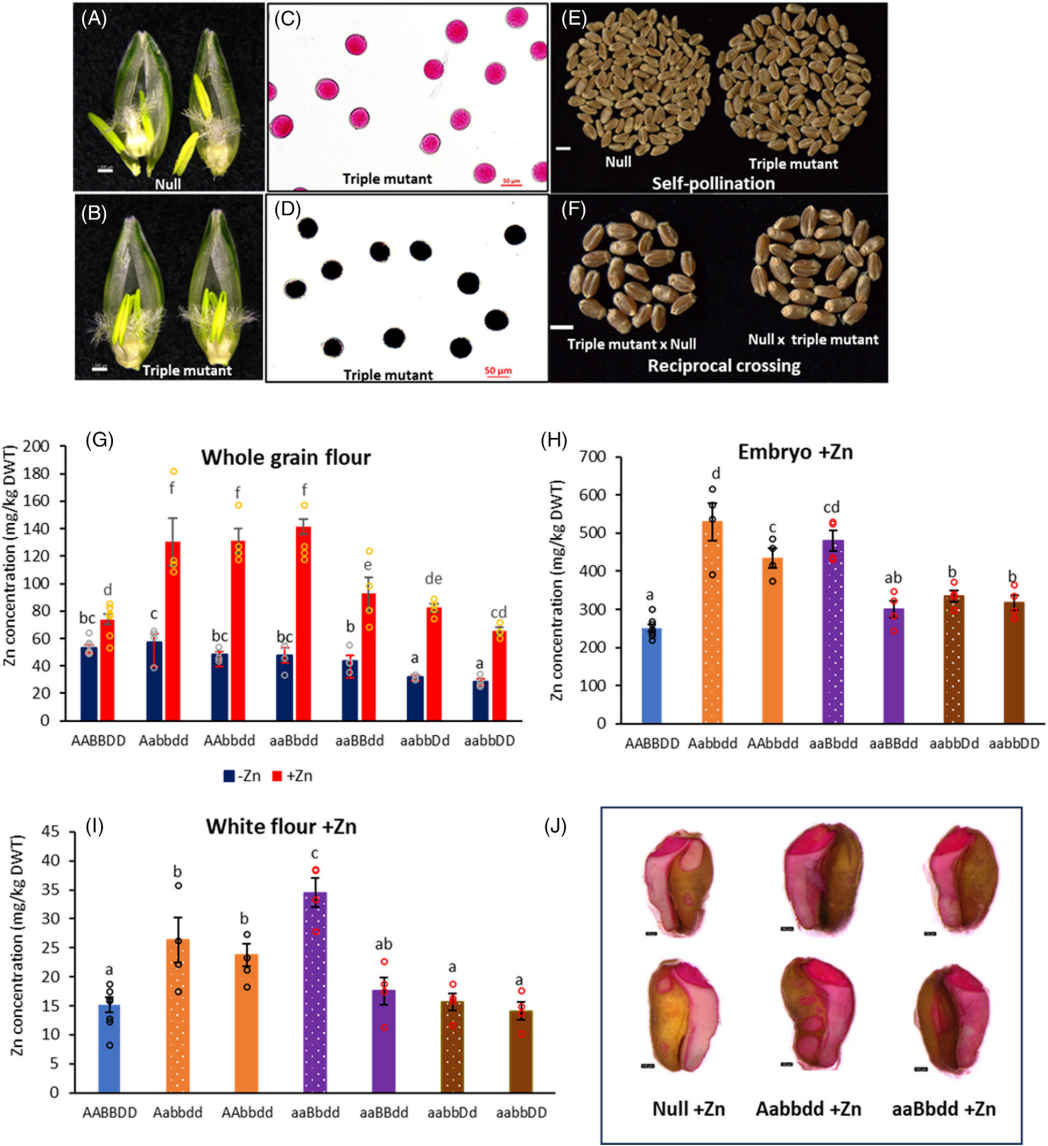
Restoration of fertility in triple mutant plants under low Zn conditions and Zn concentrations in partial mutant grains at the mature stage grown. (A–F) Triple mutants and nulls were grown in 50% sand + 50% perlite mix (by volume) watered with 0.005 μM ZnSO_4_ in nutrient solution. The triple mutants restored their fertility. (A) normal floret of null. (B) Floret of triple mutant restored fertile phenotype. (C) pollen restored to viability with complete cytoplasm and (D) full of starch. (E) Seeds from self‐pollination of null and triple mutant. (F) Seeds from reciprocal cross‐pollination of null ovules with triple mutant pollen and triple mutant ovules with null pollen. Scale bars represent 1000 μm in (A, B), 50 μm in (C, D), and 5 mm in (E, F). (G–J) Zn concentrations in partial mutant grains at mature stage grown in compost with 100 mg ZnSO_4_·7H_2_O application in soil mix at the potting stage and 100 mg ZnSO_4_·7H_2_O in solution per pot at the anthesis stage. Zn concentrations were measured using ICP‐OES. (G) Zn concentrations in whole grain flour under −Zn and +Zn conditions. (H) Zn concentration in embryo from +Zn plants. (I) Zn concentration in white flour (mainly central starch endosperm) milled from de‐embryoed grains under the +Zn condition using a mini mill. Data are means ± SE from eight biological replicates (*n* = 8) for two combined nulls (AABBDD) and four biological replicates (*n* = 4) for mutants. Different letters indicate significant differences as determined by two‐way multistratum ANOVA in (G) or one‐way multistratum ANOVA in (H, I) followed by an LSD test for mean comparisons (*P* < 0.05). (J) Mature wheat grains were stained using dithizone in methanol for Zn concentration. Scale bars represent 100 μm.

The triple mutants were subsequently grown in a 50% sand + 50% perlite mixture (by volume), with 0.005 μM ZnSO_4_ provided until maturity. The triple mutants had restored fertility and grew normally, comparable to the nulls, and there were no differences in the florets, anthers, carpels (Figure 6A,B), ear length and morphology (Figure S7h,j), plant height (Figure S7i) between the triple mutants and nulls. Pollen viability was confirmed through staining (Figure 6C,D) and showed no difference from null (Figure S7f,g). To verify fertility, two reciprocal crosses (triple mutant (♀) × null (♂) and null (♀) × triple mutant (♂)) produced 76–89% seeds, and self‐pollinated triple mutants produced seeds at the same rate as the nulls (Figure 6E,F). The grain Zn concentrations were low, with levels of 16.12 mg/kg and 14.74 mg/kg for the null and mutant types, respectively, showing no significant difference (Figure S7k). The KASP assay confirmed that the BC_3_F_4_ plants from the self‐pollinated seeds were triple mutants and showed the same phenotypes under −Zn and +Zn conditions as the previous generation BC_3_F_3_.

### Grain Zn concentrations were affected by Zn conditions and genotypes in partial mutants

Partial mutants (Aabbdd, AAbbdd, aaBbdd, aaBBdd, aabbDd, and aabbDD) exhibited no observed phenotypic differences from nulls in fertility, growth, or flowering time (Figure S8a–d), indicating that a single wild‐type allele is sufficient to maintain fertility.

To assess whether grain Zn concentrations were influenced by genotypes and Zn availability, six mutant genotypes at the BC_3_F_3_ generation (Aa/AAbbdd, aaBb/BBdd, aabbDd/DD) were developed from BC_3_F_2_ seeds of Aabbdd, aaBbdd, and aabbDd plants. These plants were then grown under both −Zn and +Zn conditions. No substantial differences were observed between nulls and mutants in plant height, ear length, grain number per plant, or grain weight per plant, except that aabbDd/DD plants were slightly taller, while Aa/AAbbdd and aaBb/BBdd exhibited a slight reduction in ear length and grain number per plant (Figure S8e–h).

However, grain Zn concentrations in whole grain flour were significantly affected by both genotypes and Zn conditions (Figure 6G). Under −Zn conditions, grain Zn concentrations were similar between nulls and mutants except aabbDd/DD with a slight reduction. In contrast, under +Zn conditions, some mutants (Aa/AAbbdd, and aaBbdd) exhibited significantly higher grain Zn concentrations (1.75 to1.90‐fold increase) compared with nulls (Figure 6G). Zn concentrations were significantly increased in both the embryo from these mutants (Aa/AAbbdd, aaBbdd, aabbDd/DD) (Figure 6H) and white flour from these mutants (Aa/AAbbdd, and aaBbdd) (Figure 6I) with grains being strongly stained in embryo and endosperm (Figure 6J).

To determine which vegetative organs contributed to the higher Zn concentrations in the mutant grains, Zn concentrations throughout the plant parts were analyzed using ICP‐OES. The Aa/AAbbdd and aaBb/BBdd mutants exhibited significantly lower Zn concentrations and distributions in the stems (the axis below the flag leaf node) and the flag leaf node (Figures S9a,c and S10a,b), but higher Zn concentrations and distributions in the grain (Figure 6G–J, Figure S10f,g). These findings suggest that in the Aa/AAbbdd and aaBbdd mutants under +Zn conditions, more Zn is translocated from the stem to the grain during the grain‐filling stage. Among the tested genotypes, aabbDd/DD had the least impact on grain Zn concentration, while Aa/AAbbdd and aaBbdd exhibited the strongest effect.

## DISCUSSION

### Functional validation of TaMTP1 as a tonoplast‐localized Zn transporter

Functional characterization of TaMTP1 in yeast and subcellular localization support its role as a vacuolar membrane Zn transporter. The ability of TaMTP1 to rescue the Zn‐sensitive phenotype in yeast lacking Zn sequestration mechanisms highlights a conserved function for the plant transporter. This is consistent with previous findings in rice (*OsMTP1*) (Menguer et al., 2013), *Triticum urartu* (*TuMTP1*) (Wang et al., 2018), and barley (*HvMTP1*) (Podar et al., 2012). The upregulation of *TaMTP1* under high Zn conditions represents a typical response of metal detoxification genes and indicates that its transcription is tightly regulated by Zn status, a central aspect of Zn homeostasis. Together, these results suggest that TaMTP1 facilitates Zn detoxification by mediating Zn sequestration into vacuoles, thereby mitigating cytosolic toxicity. Future transcriptomic and promoter analyses will be valuable for identifying upstream regulators and Zn‐responsive transcription factors that modulate *TaMTP1* expression under varying Zn regimes.

In hexaploid wheat, functional redundancy among homeologs at syntenic loci is a common feature of essential genes involved in nutrient homeostasis. TaMTP1A, TaMTP1B, and TaMTP1D exhibit exceptionally high amino acid sequence identity and fully conserved domain architecture, strongly supporting functional equivalence among the three homeologs. In this biological context, functional characterization of TaMTP1A provides a valid proxy for the transport activity and subcellular localization of all three TaMTP1 homeologs. This interpretation is consistent with the severe fertility defects observed in the TaMTP1A/1B/1D triple mutant, as discussed in detail below.

### Zn hyperaccumulation in triple mutant reproductive organs impaired wheat fertility and growth

The generation of triple knockout mutants provided insights into the essential function of *TaMTP1* in Zn homeostasis and plant fertility. These mutants exhibited severe developmental defects, including sterility, reduced plant height, shortened ear and delayed flowering under elevated Zn conditions. ICP‐OES data and fluorescence imaging revealed excess Zn accumulation in reproductive organs (anther, carpel and ear) of triple mutants, leading to cytosolic toxicity. This disrupted gametophyte development and impaired starch deposition, likely due to failed vacuolar Zn sequestration. Importantly, rescue of fertility and normal growth in triple mutants under low Zn conditions further confirmed that Zn toxicity, rather than inherent developmental defects, drives these Zn‐hypersensitive phenotypes. However, the sterility is observed only in triple homozygous mutants (aabbdd), whereas partial mutants harboring a single wild‐type *TaMTP1* allele (Aabbdd, aaBbdd, or aabbDd) retained full fertility, showing no phenotypic difference from nulls. This suggests the polyploid nature of hexaploid wheat confers functional redundancy and adaptation among the three homeologs during evolution.

Unlike *hma2/hma4 or mt2b/mt2c* double mutants, where sterility or reduced fertility typically arose from Zn deficiency in reproductive tissues (Hermand et al., 2014; Hussain et al., 2004; Lei et al., 2021), *tamtp1* triple mutants exhibited Zn toxicity‐induced sterility. The associated plant growth inhibition symptoms (including reduced plant height, spike length, anther and pollen size) may be attributed to Zn‐induced suppression of auxin synthesis (Wang et al., 2021), or reactive oxygen species (ROS) overaccumulation (Kawachi et al., 2009; Sankaranarayanan et al., 2020), both known to impair cell division and gametophyte development. Investigation of ROS accumulation and antioxidant responses in *tamtp1* triple mutants in future studies will further clarify the mechanistic link between *TaMTP1* loss‐of‐function, Zn dyshomeostasis, and reproductive defects.

Interestingly, reproductive defects have not been reported in *mtp1* mutants of Arabidopsis and rice (Desbrosses‐Fonrouge et al., 2005; Ning et al., 2023), indicating functional divergence and specification of *TaMTP1* in wheat during evolution despite high sequence conservation (Vatansever et al., 2017).

### Mechanism of *TaMTP1‐*mediated Zn allocation from stem to spike via vacuolar sequestration

**Figure 7:**
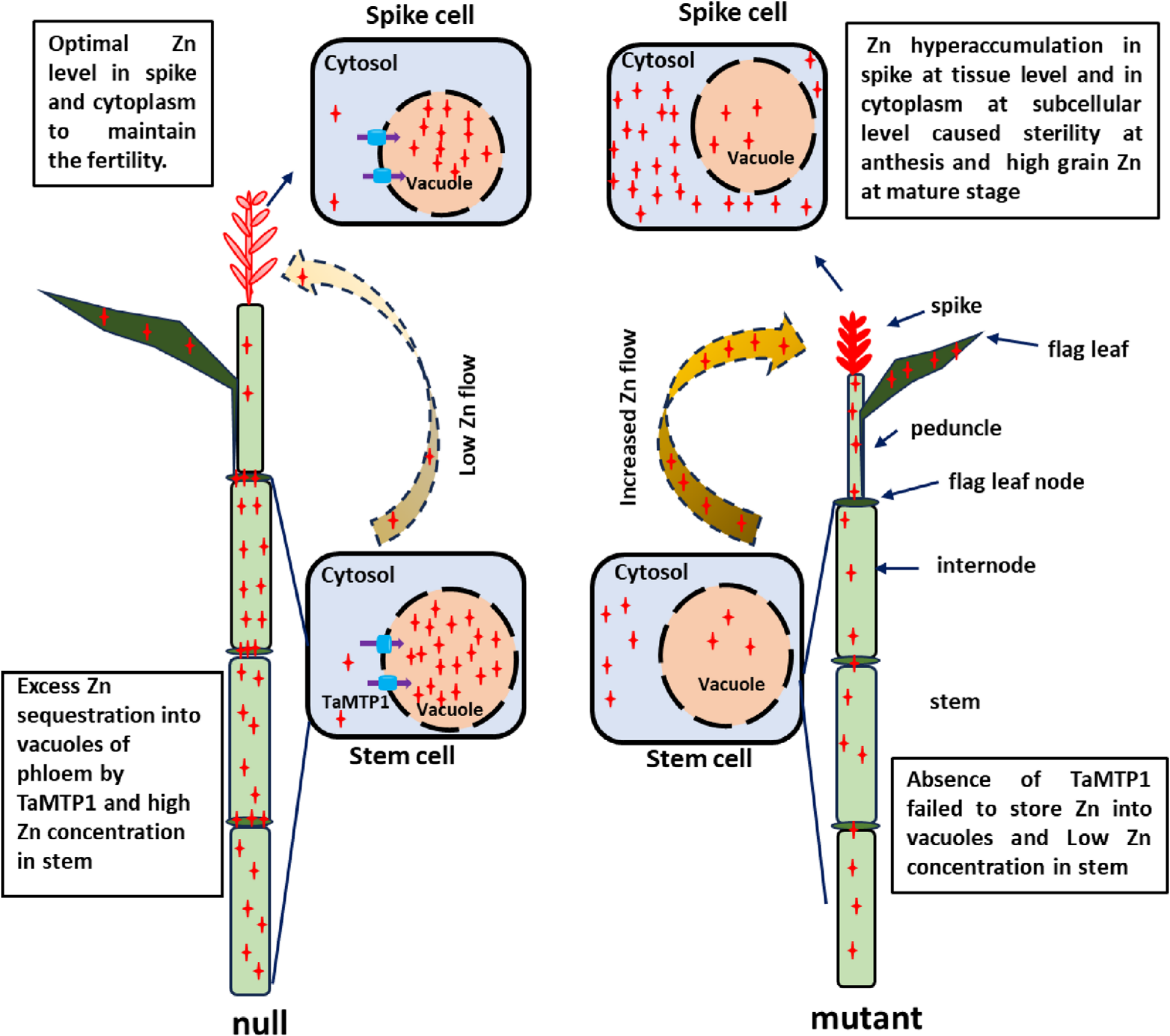
Proposed model of *TaMTP1*‐mediated vacuolar Zn sequestration and the impact on Zn allocation in wheat. In null plants (left), TaMTP1 localizes to the tonoplast membrane and sequestrates excess Zn into vacuoles of phloem cells in the stem, thereby reducing cytosolic Zn and limiting its translocation to spike to ensure fertility. This results in higher Zn retention in the stem. In contrast, *TaMTP1* knockout plants (right) failed to store Zn into vacuoles, likely leading to elevated cytosolic Zn that may be more readily mobilized to reproductive tissues, reducing Zn concentration in the stem and increasing its allocation to the spike. TaMTP1 transporter: 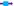; Free Zn: 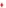.

Reproductive organs require Zn during anthesis and grain filling, but Zn allocation must be tightly regulated to avoid toxicity. In this study, elemental analysis showed that Zn supplementation did not alter Zn concentrations in the reproductive organs (anthers, carpels or ears) of null plants, suggesting a protective mechanism that maintains fertility. Instead, excess Zn accumulated in vegetative tissues (stem, leaf, and peduncle) at anthesis. In contrast, triple mutants exhibited Zn hyperaccumulation in reproductive organs but showed a decrease in stem Zn concentration under +Zn conditions, especially in the nodes. The fluorescence imaging further confirmed decreased Zn sequestration in the nodal phloem cells of enlarged, diffuse vascular bundles, and in phloem and xylem parenchyma cells of the internode in mutants. Additionally, high *TaMTP1* expression in stems and spikes supports its role in vacuolar Zn sequestration and detoxification at these sites. Together, these findings support a model that TaMTP1 maintains fertility by modulating Zn translocation from stems to spikes via vacuolar sequestration, with the stem nodes acting as the primary regulatory site (Figure 7). In the grain, *TaMTP1* expression in the embryo and aleurone (Figure 2D, Figure S11c) also suggests a role in subcellular Zn partitioning during seed development.

In triple mutants, the absence of TaMTP1 disrupted Zn sequestration into vacuoles in the stem, likely leading to elevated cytosolic Zn, which may subsequently increase Zn movement toward the reproductive organs due to their high Zn demand. Nodes act as a valve controlling Zn loading from stem to spike. Consequently, excess Zn accumulation in the spike caused tissue‐level toxicity. Furthermore, failed vacuolar Zn sequestration in reproductive organs led to subcellular toxicity, impairing pollen, carpel, and young spike development, ultimately resulting in sterility and reduced spike length. Conversely, TaMTP1 in wild‐type plants facilitates vacuolar Zn compartmentalization in stem phloem cells, limiting Zn translocation to spikes and thereby protecting reproductive development and ensuring normal fertility.

Further validation of this model will require direct quantification of intracellular Zn pools and Zn transport dynamics. Future experiments using genetically encoded FRET‐based Zn sensors (e.g., eCALWY) (Lanquar et al., 2014) could visualize real‐time changes in cytosolic Zn^2+^ concentrations, while xylem and phloem sap elemental profiling would help quantify Zn fluxes through the stem–spike pathway. In addition, transcriptomic analyses of Zn‐related transporters could clarify how the loss of TaMTP1 reshapes Zn homeostasis networks. Similar protective roles of vacuolar metal sequestration in maintaining reproductive viability have been demonstrated for phosphate transporters AtVPT1/AtVPT3 in Arabidopsis (Luan et al., 2019) and the Cd transporter OsHMA3 in rice (Yan et al., 2024), highlighting a conserved strategy across species. Overall, this work uncovers a novel Zn‐regulatory mechanism in wheat, demonstrating that TaMTP1 maintains fertility by mediating vacuolar Zn sequestration in the stem to modulate Zn delivery to reproductive organs.

### TaMTP1 enhances grain Zn concentration in partial mutants via stem‐to‐grain partitioning

In addition to protecting reproductive development, TaMTP1 also modulates Zn allocation to developing grains. Partial mutants (Aa/AAbbdd, aaBbdd) accumulated significantly higher Zn in grains, especially in embryos, and starchy endosperm under high Zn supply compared with nulls without showing major growth defects. This indicates that loss of both *TaMTP1*B and *TaMTP1*D expression enhanced Zn translocation from stems to grains in a dosage‐dependent manner, likely due to their predominant expression in stems and spikes. Expression analyses showed that *TaMTP1B* accounted for 39–47% of total *TaMTP1* expression in the shoot axis, 35–39% in internodes, and 36–41% in the flag leaf node, while *TaMTP1D* contributed 33–37% in the shoot axis, 40–46% in internodes, and 32–36% in the flag leaf node, respectively. In contrast, *TaMTP1A* contributed only 19–26% in the shoot axis, 19–22% in internodes, and 23–29% in the flag leaf node (Figure S11a,b). These results reveal distinct functional contributions of the three *TaMTP1* homeologs. Future transcriptomic analyses under different Zn regimes could elucidate potential regulatory hierarchies and homeolog compensation mechanisms among *TaMTP1A*, *TaMTP1B*, and *TaMTP1D*. Similar gene dosage effects were reported for five *AhMTP1* paralogs in *Arabidopsis helleri* (Zn hyperaccumulator), which showed divergent contributions to Zn tolerance (Shahzad et al., 2010).

The underlying mechanism is consistent with that regulating spike fertility: reduced vacuolar sequestration in stems increases Zn flux to developing grains. Unlike rice OsMTP1 and OsHMA3, which modulate root‐to‐grain Zn and Cd partitioning (Ning et al., 2023; Yan et al., 2024), and OsVIT2, which mediates node 1 and leaf sheath‐to‐grain translocation (Che et al., 2021), TaMTP1 primarily regulates stem‐to‐grain translocation via vacuolar sequestration. Moreover, elevated Zn accumulation in wheat grains primarily occurred in both embryos and starchy endosperm, differing from rice *OsMTP1* mutants, where Zn is also redistributed from aleurone and embryo to endosperm. This highlights a distinct mechanism in wheat for controlling Zn enrichment in grains. Future comparative studies among wheat genotypes, or related species, with contrasting grain Zn levels could further clarify how natural variation in *TaMTP1* expression contributes to Zn accumulation in wheat grains, potentially leading to the discovery of beneficial alleles for breeding.

### *TaMTP1* as a target for wheat biofortification

Targeting *TaMTP1* expression offers a promising strategy to enhance Zn accumulation in wheat grains for improved nutritional quality. Previous studies demonstrated that endosperm‐specific expression of *HvMTP1* in barley resulted in increased zinc content in grains, while *AtMTP1* overexpression in cassava root elevated Zn concentration in tubers (Gaitán‐Solís et al., 2015; Menguer et al., 2018). In wheat, mutant genotypes (Aa/AAbbdd and aaBbdd) accumulated more Zn in whole grain and white flour under elevated Zn supply, demonstrating a practical potential for enhancing grain nutritional value. These findings have significant implications for biofortification strategies to combat Zn deficiencies in human diets.

## CONCLUSION

This study demonstrates that TaMTP1 plays a pivotal role in Zn homeostasis by mediating vacuolar Zn sequestration, which is essential for protecting reproductive development, maintaining fertility, and regulating grain Zn allocation in wheat. *TaMTP1* expression is finely tuned to optimize Zn allocation between stem and reproductive organs, ensuring fertility and controlling grain Zn concentration through vacuolar sequestration. These findings provide valuable insights into the mechanisms of Zn homeostasis in wheat and offer potential strategies for improving Zn tolerance and biofortification in Zn‐contaminated environments.

## MATERIALS AND METHODS

### Triple mutant generation and backcrossing

Three single mutation lines Ca0735 (*MTP1a*), Ca0178 (*MTP1b*), and Ca04039 (*MTP1d*) were selected from a hexaploid wheat (*Triticum aestivum* cv Cadenza) TILLING population, and sequentially crossed to produce triple heterozygous plants (AaBbDd). These heterozygous plants were backcrossed with wild‐type Cadenza (used as the female parent) three times, resulting in the MTP1 BC_3_F_3_ generation, as outlined in Figure S2a. Two null lines segregated as siblings with the triple mutants at the BC_3_F_2_ generation. Due to sterility and the low probability (1/64) of obtaining triple mutants in BC_3_F_2_ plants, which were insufficient for experiments, BC_3_F_3_ triple mutant plants were derived from seeds of BC_3_F_2_ plants with genotypes, such as Aabbdd, aaBbdd, or aabbDd. These plants only produced approximately 3–4% (not 25%) triple mutants at the BC_3_F_3_ generation, which were subsequently used for the experiments.

### Genotyping

Single mutations were validated by sequencing the flanking region of the mutation site amplified with genome‐specific primers, and the mutants in subsequent generations were identified using Kompetitive allele‐specific PCR assays (KASP) with FAM and VIC probe sequences to the 5′ end of the wild‐type‐ and mutant‐allele‐specific KASP primers, respectively. Primers are listed in Table S1.

Genomic DNA was extracted from freeze‐dried wheat seedling leaf samples, which were ground in deep 96‐well plates with 3 mm stainless steel balls using the Geno/Grinder 2010 (SPEX Sample Prep, Metuchen, NJ, USA), followed by incubation in extraction buffer (100 mM Tris Base, 1 M KCI, and 10 mM EDTA, pH 9.5) at 65°C for 1 h. About 3 M potassium acetate was added to remove the cell debris, and DNA was precipitated with isopropanol. After washing with 75% (v/v) ethanol, gDNA was dissolved in 10 mM Tris (pH 7.5) with RNAse.

KASP assays for high‐through genotyping were performed in an ABI‐7500 real‐time PCR system (Applied Biosystems, Waltham, MA, USA) using a PACE Low‐ROX mix (3CR Bioscience, Harlow, UK). The reaction and PCR conditions were as described by Sigalas et al. (2024). The PCR cycling parameters used were 15 min at 94°C, 10 cycles at 94°C for 20 sec and 62 or 63°C (− 0.6°C per cycle) for 60 sec, then 36 cycles at 94°C for 20 sec and 56°C or 57°C for 60 sec, and followed by final plate reading at 30°C for 60 sec.

### Growth and Zn treatment of triple mutants

The triple mutants and null lines were grown in a glasshouse under controlled conditions with a 16‐h/8‐h day‐night cycle at 20°C/16°C and 50–70% humidity. Plants were cultivated in compost soil containing Zn concentration (40 mg/kg dry weight) under two conditions: without ZnSO_4_.7H_2_O (−Zn) and with ZnSO_4_.7H_2_O supplementation (+Zn). For the +Zn treatment, 100 mg of ZnSO_4_·7H_2_O was mixed into the compost at potting, with an additional 100 mg ZnSO_4_·7H_2_O applied per pot, in solution, at the flag leaf stage.

### Experiment design and tissue harvesting in the triple mutants at anthesis

Due to limited availability of triple mutants and the narrow developmental window at anthesis, two similar experiments (Experiments 1 and 2) were sequentially conducted at anthesis. Each triple mutant line consisted of 10 individual plants derived from the same BC_3_F_2_ generation genotype (Aabbdd, aaBbdd, or aabbDd), divided equally between the two Zn treatments: 5 plants for −Zn and 5 plants for +Zn, serving as five biological replicates per treatment. In total, 20 triple mutant plants (two triple mutant lines) and 20 null plants (two null lines) were statistically randomized within five complete blocks of eight plants to minimize experimental bias.

Experiment 1 focused on anther length, anther and carpel Zn concentration, pollen viability, pollen area, and plant height analyses. Anthers and carpels were collected approximately 24–48 h before anthesis from the first and second florets of each spikelet, sampling 3–4 ears per plant. Samples were kept on ice and imaged immediately using a Leica M205 microscope. Anther lengths (30–207 anthers per plant) were measured and averaged using ImageJ software. The collected anthers and carpels were stored at −80°C for freeze drying and measured using inductively coupled plasma optical emission spectroscopy (ICP‐OES). Plant height was measured and averaged for all the tillers of each plant.

Experiment 2 examined Zn distribution among different plant organs. At anthesis, the whole ear (including anthers, carpels, rachis, glume, lemma), peduncle (including flag leaf node 1), the remaining stem, and leaf (leaf blade and sheath) from 3 to 4 tillers each plant were harvested and freeze‐dried separately for ICP‐OES analysis.

Experiment 3 aimed to assess Zn concentrations in specific stem segments, including flag leaf node 1, node 2, internode 1, internode 2, and peduncle. For this experiment, one triple mutant line and one null line were selected, each with three biological replicates under both −Zn and +Zn conditions. Samples were harvested from 3 to 4 tillers and pooled per plant for ICP‐OES after freeze drying and milled using a centrifuge miller.

### Pollen viability assay

Fresh mature pollen from yellow anthers, approximately 24 h before anthesis, was dusted on the glass slides with one drop of either Alexander's solution for cytoplasmic content staining or I_2_‐KI solution for staining of starch content. After staining for a few minutes, the pollen samples were visualized under a Zeiss Axio imager light microscope (Zeiss, Germany). For Alexander staining, the viable pollen grains were stained red or pink with full cytoplasmic content, while the non‐viable pollen grains were also stained red with partial cytoplasm (scarce pollen) or the pollen grains were unstained if lacking cytoplasmic content (empty pollen). For starch staining, pollen grains were either stained black when full of starch (viable) or with no staining indicating no starch content (non‐viable). Ten views of each sample for each staining type were captured for pollen area measurement and calculation of viable pollen percentage. Pollen diameter was measured using ImageJ software and converted to cross‐sectional area.

*In vitro* pollen germination was performed following the method described by Impe et al. (2020). Fresh pollen from yellow anthers was dispersed onto a solid medium containing 594 mM raffinose, 0.81 mM H_3_BO_3_, and 2.00 mM CaCl_2_ at pH 5.8, with 0.3% Gelrite. The samples were then incubated in a humid environment at 23°C for 3 h. The image was taken with the Leica M205 microscope.

### Scanning electron microscopy of pollen and stigma

Fresh anther and carpel samples were taken from the wheat plants and oriented onto the carbon stubs. Once the microscope reaches the set cryo temperatures, the samples were plunge‐frozen into liquid nitrogen. The frozen samples were coated with gold nanoparticles (5–10 nm) for a set time. The samples were then transferred to the JEOL 6360 SEM chamber and imaged under the accelerating voltage of 15 KV.

### Zn chemical staining and fluorescence localization in triple mutants

The fresh anthers and carpels (more than 10 samples) were stained with dithizone (500 mg/L) in methanol for 4 h. The mature grains (12 grains) were embedded in the water for 4 h, then were cut longitudinally and stained for 30 min. After being rinsed in water, the samples were visualized under the Leica M205 stereomicroscope.

For Zn localization, fresh anthers were embedded in optimal cutting temperature (O.C.T) compound (Tissue‐Tek) at −17°C and sectioned at 30 μM thickness using a cryostat (Leica CM1850). The sections were dried on poly‐L‐lysine adhesion slides overnight at 37°C and were immersed in working solution (20 μM) of Zinpyr‐1 fluorogenic Zn^2+^ reporter (Abcam ab145349) for 3 h (Sinclair et al., 2007). At least eight anthers were used for each experiment. For internodes and nodes, the sections (<0.5 mm) from three different stems were manually cut and were immediately incubated in Zinpry‐1 working solution for 3 h. After incubation, the sections were rinsed with deionized water and visualized under a Leica confocal Stellaris 8 Falcon microscope at 488 nm excitation. 10 mM PBS (phosphate‐buffered saline) buffer was used instead of Zinpyr‐1 working solution as negative controls (Figure S7). The experiment was conducted using eight anthers per replicate and repeated twice for consistency.

### Triple mutants were grown in low Zn soil to restore its fertility

In Experiment 4, low fertility soil consisting of loamy sand with minimal organic content and Zn concentration 20 mg Zn/kg dry weight was purchased from Bourne Amenity Ltd, UK. This soil was used for experiment requiring reduced Zn levels for fertility recovery of triple mutants. Nutrient solutions without Zn supplementation were applied at three key growth stages: tillering, anthesis, and grain filling. Three null plants and three triple mutants were examined for fertility.

In Experiment 5, 50% sand +50% perlite (by volume) mixed medium was supplemented with 0.005 μM ZnSO_4_ (a low level of Zn) to restore fertility and plant growth of triple mutants. The triple mutants identified through KASP genotyping were washed to remove soil and transferred to this growth medium. Plants were supplied with a nutrient solution containing the following components: macronutrients: 1.5 mM Ca(NO_3_)_2_, 5 mM KNO_3_, 2 mM NaNO_3_, 1 mM MgSO_4_, 0.5 mM KH_2_PO_4_; micronutrients: 25 μM FeEDTA, 0.2 μM CuCl_2_, 1 μM H_3_BO_3_, 0.6 μM MnCl_2_, 0.1 μM Na_2_MoO_4_, 5 μM KCl, all buffered with 2.5 mM 2‐(N‐morpholino) ethanesulfonic acid monohydrate (MES), pH 5.8, and Zn Supplementation: 0.005 μM ZnSO_4_. The nutrient solution was applied three times per week (Monday, Wednesday, and Friday) until the plants reached maturity. Three null plants and three triple mutants were grown in this growth medium.

Reciprocal crosses were performed between triple mutants and null plants. Three ears from three different triple mutants were emasculated and hand‐pollinated with pollen from null plants. Null plant ears were similarly emasculated and hand‐pollinated with pollen from triple mutants. To prevent contamination, the emasculated and pollinated ears were covered with plastic bags.

### Partial mutant growth and Zn allocation to grains at mature stage

In Experiment 6, six genotypes of partial mutants (Aabbdd, AAbbdd, aaBbdd, aaBBdd, aabbDd, and aabbDD) segregated with triple mutants at the BC_3_F_3_ generation in Experiment 2 and two null lines were selected for a comparative analysis of Zn allocation to grains at mature stage. A total of 64 plants were grown under two Zn conditions: −Zn (no added ZnSO_4_·7H_2_O) and +Zn (100 mg of ZnSO_4_·7H_2_O) mixed into the compost at potting, with an additional 100 mg ZnSO_4_·7H_2_O applied in solution at anthesis. Treatments (eight lines × two Zn conditions) were randomized according to a complete block design with four blocks of 16 plants.

Individual tissues, including leaves (with sheath), peduncle, stem, flag leaf node (Node 1), and ear, were harvested. The samples were oven‐dried at 80°C overnight, weighed, and milled into a fine powder using a centrifugal mill (Retsch, ZM200, Germany). These milled tissue samples were analyzed for Zn concentrations using ICP‐OES.

For embryo and white flour preparation, approximately 4 grams of mature grains (10.5% moisture content) were conditioned to 15.5% moisture overnight. The embryos were manually removed using a scalpel. The embryo‐excised grains were cut longitudinally along the groove and milled using a Metefem mini mill (Italy). The flour was sieved through 250 μm and 150 μm sieves to obtain three fractions: mainly bran, coarse flour, and fine white flour. The white flour and embryos are measured for Zn concentrations using ICP‐OES.

### Elemental analysis using ICP‐OES

The milled or freeze‐dried samples were oven‐dried at 80°C overnight, and digested using a mixture of nitric acid and perchloric acid (85:15 v/v) in open tube digestion blocks, followed by a programmed heating digestion: 60°C for 180 min, 100°C for 60 min, 120°C for 60 min, 175°C for 90 min and 50°C until dry as described by Wan et al. (2022). The elements were detected with Optima 7300 DV Inductively Coupled Plasma‐Optical Emission Spectrometer (ICP‐OES).

### *TaMTP1* expression in yeast and yeast complementation assay

Full length of *TaMTP1A* gene was cloned into pGEM‐T Easy vector using primers listed in Table S1. After sequencing, *TaMTP1A* was subcloned into vector pYES2, and transformed into wild‐type yeast (*Saccharomyces cerevisiae*) strain BY4741 (MATα; his3▵1; leu2▵0; met15▵0; ura3▵0) from Euroscarf, Frankfurt, Germany and the mutant strain zrc1/cot1 (BY4741 + zrc1::natMX cot1::kanMX4) provided by Dr U. Kramer (Ruhr University, Bochum) using the S.c. EasyComp Transformation Kit (Invitrogen) according to the manufacturer's instructions. Empty vector was transformed in parallel in the yeast. Yeast induction and growth preparation was described as (Evens et al., 2017). Seven microliters of the dilutions were spotted on the plates containing 0 mM, 0.25 mM, and 1 mM ZnSO_4_, and incubated at 30°C and photographed after 7 days.

### Subcellular localization of TaMTP1

The full‐length CDS of *TaMTP1A* was cloned into the PAN580 vector containing green fluorescent protein (GFP) using Golden Gate cloning to generate the construct 35S:GFP–*TaMTP1A*. As a control, a vacuolar membrane marker construct (35S:GFP–INT1‐mKate) was prepared using the vacuolar membrane protein INT1 (inositol transporter) as a reference (Wolfenstetter et al., 2012). Both 35S:GFP–*TaMTP1A* and the control construct were introduced into wheat protoplasts by polyethylene glycol (PEG)‐mediated transformation (Yoo et al., 2007). Transformed protoplasts were incubated at 24°C for 24 h, mounted on slides, and visualized using a laser confocal microscope (Nikon C2‐ER, Japan).

### Real‐time PCR, public RNA‐seq data extraction, and hydroponic growth

RNA was extracted from 2‐week‐old wheat seedling shoots and roots grown in a controlled environment chamber, and cultured in hydroponic nutrient solution supplemented with 0.005, 0.5, 5, and 50 μM ZnSO_4_, following the method described by Sigalas et al. (2024). RNA extraction was performed using RNeasy Plant Mini Kit (Qiagen, Hilden, Germany). After DNase treatment, 2 μg of RNA was used for cDNA synthesis via reverse transcription with SuperScript™ III Reverse Transcriptase (Invitrogen) and anchored oligo (dT)_23_ primers (Sigma‐Aldrich). Real‐time PCR was conducted using an ABI 7500 thermocycler (Applied Biosystems), and normalized relative gene expression was calculated as described by Wan et al. (2021). Wheat actin3 (*TaACT3*) and succinate dehydrogenase (*TaSDH*) genes were used as reference genes, and the primer sequences were described in Table S1.

*TaMTP1* RNA‐seq data from multiple stages and tissues (Figure 1D, Figure S11a; Ramírez‐González et al., 2018) and RNA‐seq data from different grain cells (Figure S11c; Pfeifer et al., 2014) were extracted from the website (www.wheat‐expression.com), and the atlas expression image of TaMTP1A (Figure S1a) is obtained from the website: Wheat eFP Browser.

### Wax section preparation

Anther was fixed in 4% (w/v) paraformaldehyde in 0.1 M Sorenson's phosphate buffer (NaH_2_PO_4_·2H_2_O and Na_2_HPO_4_·12H_2_O, pH 7.0) containing 2.5% (w/v) glutaraldehyde (Wan et al., 2014). After filtration in vacuum and dehydration in increasing concentrations of ethanol and HistoClear, the sections were embedded in paraffin (Paraplast Plus, Sigma‐Aldrich). Wax‐embedded anther was sectioned at 14 μm thickness using a Leica JUNG Biocut 2035 rotary microtome. After HistoClear dewaxing and decreasing ethanol hydration, sections were stained using 0.5% Safranin O and 0.1% Astra Blue in 50% ethanol for 1 min.

### High‐throughput RNA‐sequencing of node 1 and data analysis

Total RNA (2 μg per sample) purified from node 1 at anthesis (0) and 7–38 days post‐anthesis (DPA) of cultivar Paragon grown in Rothamsted Research WGIN 2016 diversity trial at 200 kg N ha^−1^ fertilization treatment was used for RNA‐seq library preparation. Quality control, poly‐A selection for rRNA removal, library preparation, multiplexing and sequencing were performed by the High‐Throughput Genomics Group at the Wellcome Trust Centre for Human Genetics, Oxford University, according to their established standard procedures. Next‐generation sequencing was performed on the Illumina HiSeq 4000 platform, using a 2x150 bp pair‐end configuration. Raw paired‐end data were delivered in fastq format. Raw paired‐end reads were processed on the Rothamsted Research Galaxy platform (https://galaxy.rothamsted.ac.uk/). Adapter trimming and removal of low‐quality reads were performed prior to alignment. Raw reads were pseudo‐aligned to wheat reference transcriptome using Kallisto tool, and transcript‐level abundances were quantified as described in (Sigalas et al., 2024).

### Statistical analysis

Data were analyzed using one‐ or two‐way multistratum ANOVA incorporating nested contrasts to compare between and within null and mutant groups where appropriate. Where necessary, data were log transformed (base 10) to satisfy homogeneity of variance. Analyses were performed using Genstat (23rd edition; VSN International, Hemel Hempstead). Least significant differences (LSDs, 5% level) between means are for comparisons.

## AUTHOR CONTRIBUTIONS

YFW and MJH designed the experiment, YFW, DYZ, RT, AMU, and PPS performed the experiments, SJC did the statistical analysis, YFW and MJH drafted the manuscript, and all the authors revised the manuscript.

## CONFLICT OF INTEREST

None declared.

## Supporting information


**Figure S1.** *TaMTP1A* atlas expression and the mutation sites and amino acid sequence identity of three *TaMTP1* homeologs.
**Figure S2.** Generation and phenotypes of triple mutants.
**Figure S3.** The pollen size and viability assessment.
**Figure S4.** Minerals (Fe and Mn) in anther and carpel of nulls and triple mutants at anthesis.
**Figure S5.** Minerals in different tissues of nulls and triple mutants at anthesis.
**Figure S6.** Negative control of anther and node for Zn localization using Zinpyr‐1.
**Figure S7.** Triple mutants restored their fertility when grown under low or extremely low Zn conditions.
**Figure S8.** Phenotypes and agronomic traits of partial mutants grown in compost without ZnSO_4_.7H_2_O (−Zn) and with 200 mg ZnSO_4_.7H_2_O application.
**Figure S9.** Zn concentrations at mature stage in partial mutant plant tissues grown in compost with 200 mg ZnSO_4_.7H_2_O application.
**Figure S10.** Zn distributions at mature stage in different tissues of partial mutant plants grown in compost with 200 mg ZnSO_4_.7H_2_O (+Zn).
**Figure S11.** The contribution of homeologs in gene expression during different growth stages.

**Table S1.** Primers for KASP genotyping, yeast cloning, and qPCR.

## Data Availability

All data supporting the findings of this study are included within this article and its supporting information files. The raw data from node1 specific RNA‐seq are deposited at ArrayExpress (E‐MTAB‐15557). The link is https://www.ebi.ac.uk/biostudies/ArrayExpress/studies/E‐MTAB‐15557?key=9d283454‐aca7‐4872‐aabd‐c1ac83eb7810.
