# Supplementary material for: Vacuolar zinc transporter TaMTP1 safeguards wheat fertility and regulates grain zinc allocation via stem‐mediated sequestration
Source: Plant J. 2026 Apr 16;126(1):e70870. doi: 10.1111/tpj.70870 (PMC13086238; doi:10.1111/tpj.70870)
Supplement: Supplementary file 1 — Figure S1. TaMTP1A atlas expression and the mutation sites and amino acid sequence identity of three TaMTP1 homeologs. Figure S2. Generation and phenotypes of triple mutants. Figure S3. The pollen size and viability assessment. Figure S4. Minerals (Fe and Mn) in anther and carpel of nulls and triple mutants at anthesis. Figure S5. Minerals in different tissues of nulls and triple mutants at anthesis. Figure S6. Negative control of anther and node for Zn localization using Zinpyr‐1. Figure S7. Triple mutants restored their fertility when grown under low or extremely low Zn conditions. Figure S8. Phenotypes and agronomic traits of partial mutants grown in compost without ZnSO4.7H2O (−Zn) and with 200 mg ZnSO4.7H2O application. Figure S9. Zn concentrations at mature stage in partial mutant plant tissues grown in compost with 200 mg ZnSO4.7H2O application. Figure S10. Zn distributions at mature stage in different tissues of partial mutant plants grown in compost with 200 mg ZnSO4.7H2O (+Zn). Figure S11. The contribution of homeologs in gene expression during different growth stages. [file TPJ-126-0-s002.pdf]

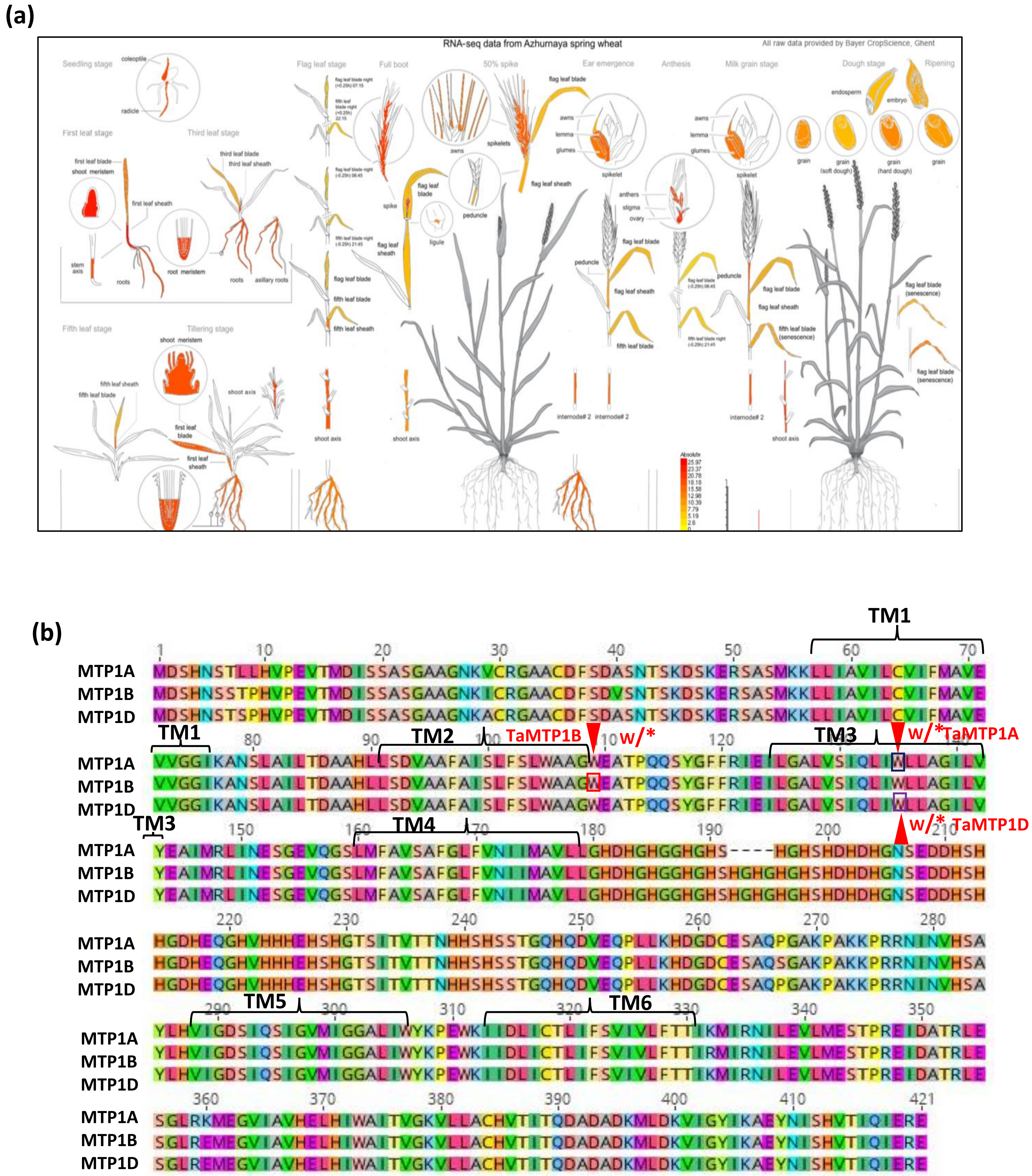

Figure S1. ***TaMTP1A* atlas expression and the mutation sites and amino acid sequence identity of three *TaMTP1* homeologs.**

(a) *TaMTP1A* expression patterns at different stages and tissues from publica RNA-seq data (Ramírez-González et al, 2018) from Wheat eFP Browser. (b) The mutation positions of three homeologs and similarity of amino acid sequences of *TaMTP1* homeologs. The red arrow heads indicated the mutation sites. The predicted transmembrane domains in TaMTP1 are denoted TM1–TM6 based on Wang et al (2018). TM: transmembrane domain.

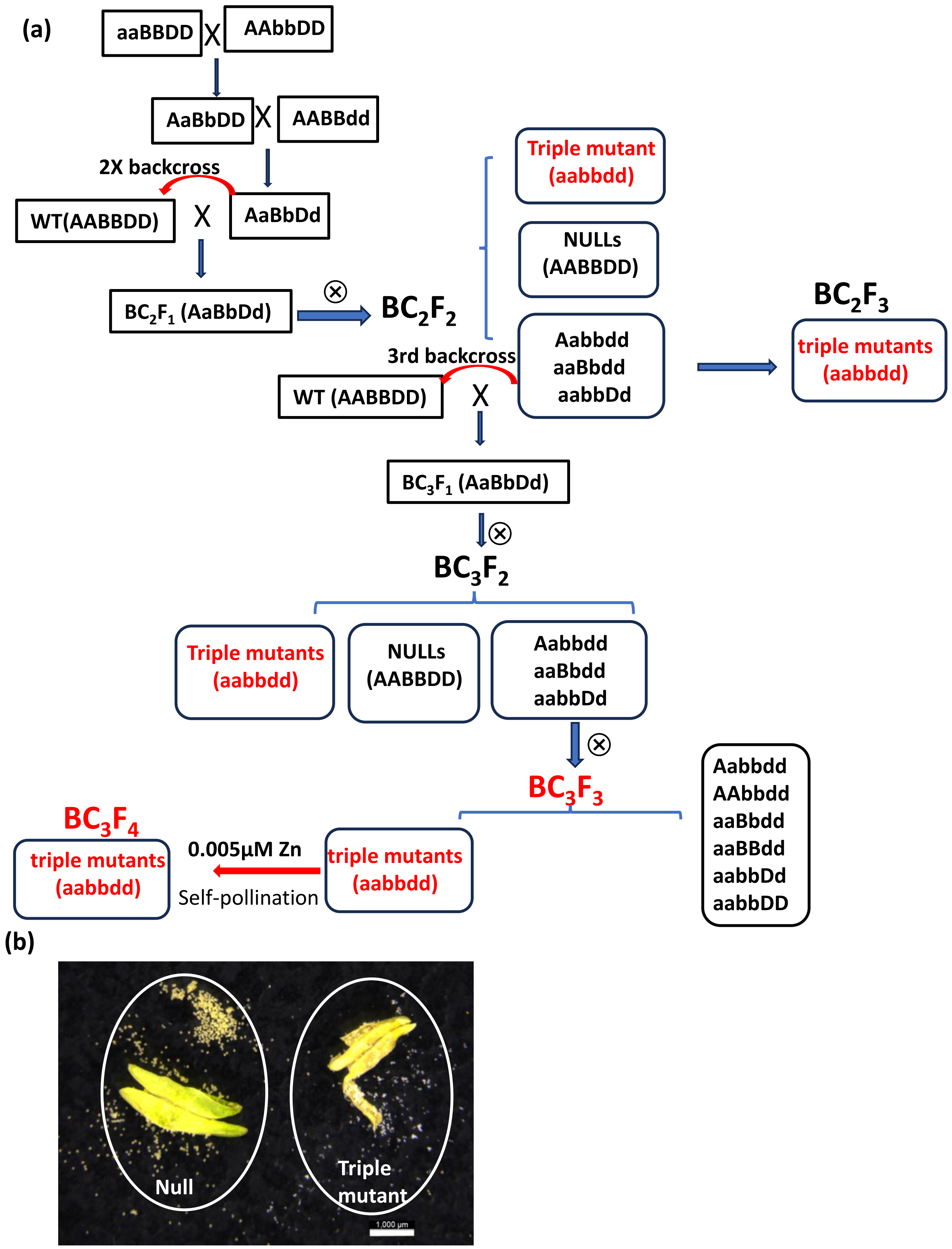

Figure S2. Generation and phenotypes of triple mutants.
(a) The triple mutants were generated by stacking three mutations and backcross to BC_3F_3 generation. The triple mutants are sterile in BC_2F_2, BC_2F_3, BC_3F_2, and BC_3F_3 when the plants grew in the compost. Only BC_3F_3 plants produced seeds when they were grown in 50% sand + 50% perlite (by volume) supplemented with extremely low Zn (0.005μM) shown in Figure 6a-f. Pollen in (b) of triple mutant and null with 200mg $ZnSO_4.7H_2O$ (+Zn). Scale bar represents 1000μm in (b). ⊗ : self-pollination

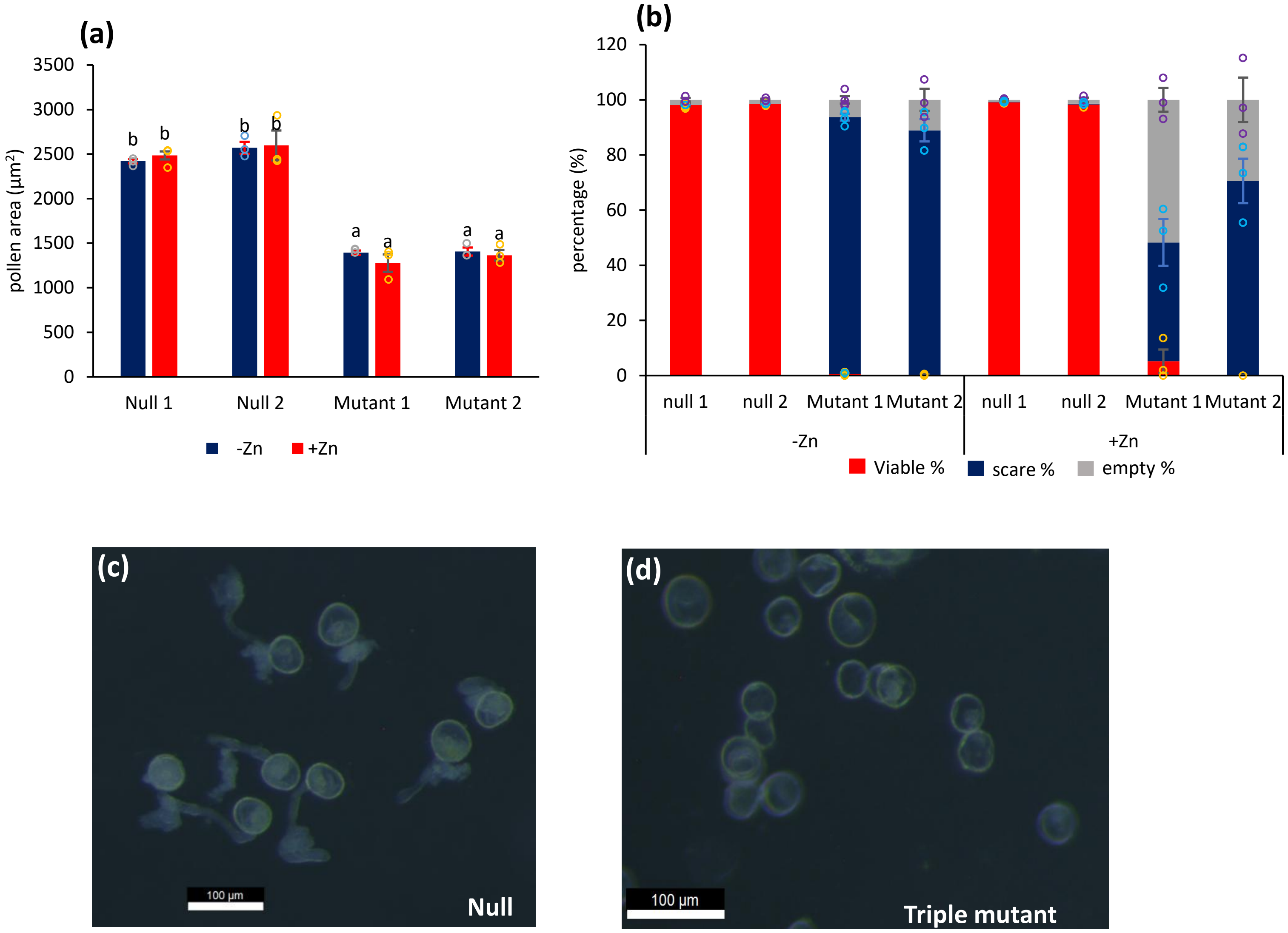

Figure S3. The pollen size and viability assessment.
(a) Pollen area of null and triple mutant grown in compost without $ZnSO_4.7H_2O$ (–Zn) and with 200mg $ZnSO_4.7H_2O$ (+Zn) application per pot. The data were analysed using an LMM/REML from three or four biological replicates (n=3-4) followed by an LSD (least significant difference) test for mean comparisons ($P < 0.05$). Error bars represent ± 1 x SE (standard error). (b) The percentage of each pollen type : viable, scarce (partial cytoplasm) or empty based on Alexander's staining (n=3). (c) pollen germination of null and (d) triple mutant on medium. Scale bar represents 100µm.

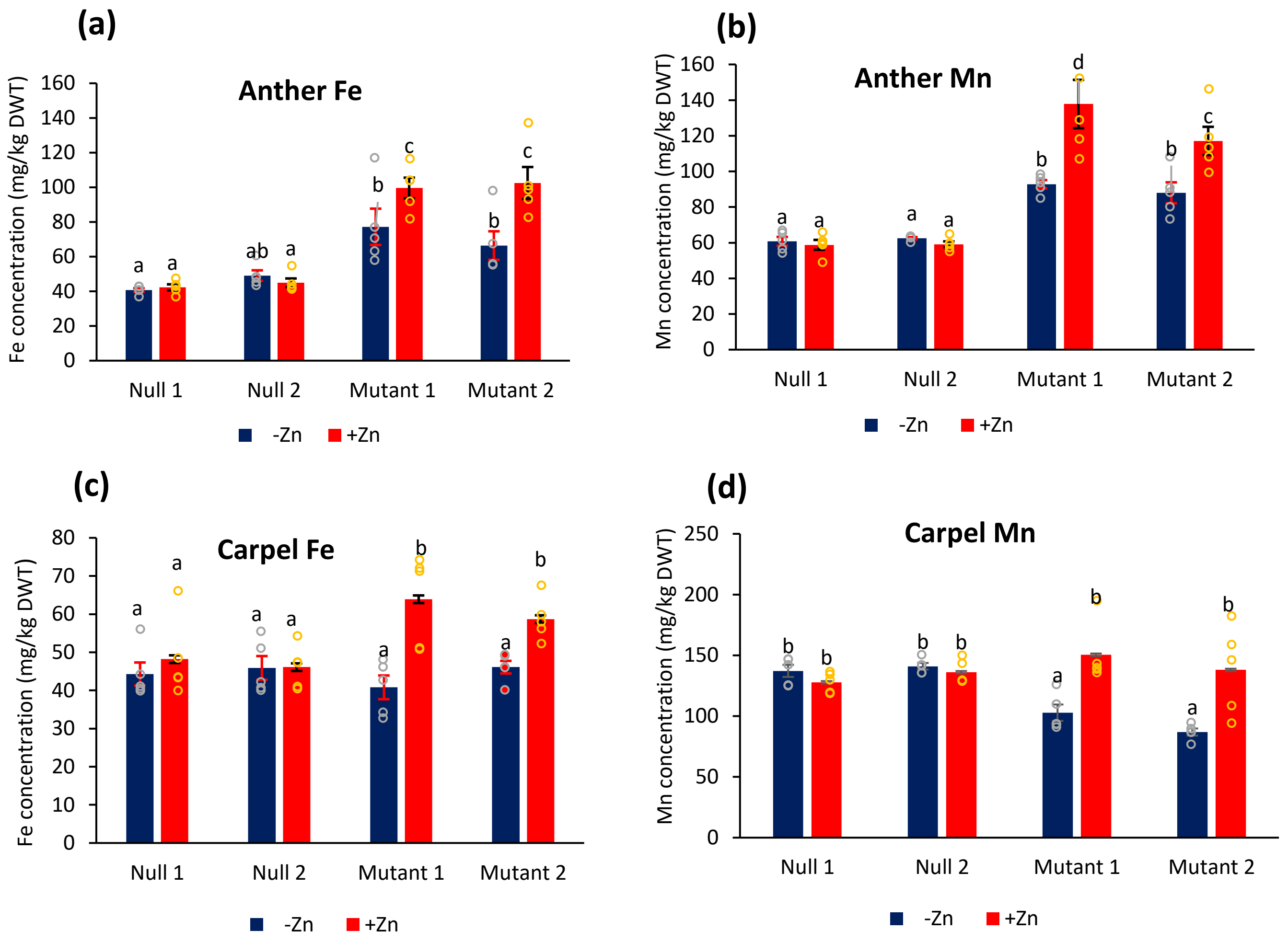

Figure S4. Minerals (Fe and Mn) in anther and carpel of nulls and triple mutants at anthesis.

Plants were grown in compost without $ZnSO_4.7H_2O$ (-Zn) and with 200mg $ZnSO_4.7H_2O$ (+Zn) application per pot, and minerals were measured using ICP-OES. (a) Fe concentration in anther. (b) Mn concentration in anther. (c) Fe concentration in carpel. (d) Mn concentration in carpel. Data were analysed using two-way multi-stratum ANOVA (n=5) followed by an LSD test for mean comparisons ($P < 0.05$). Error bars represent ± 1 x SE .

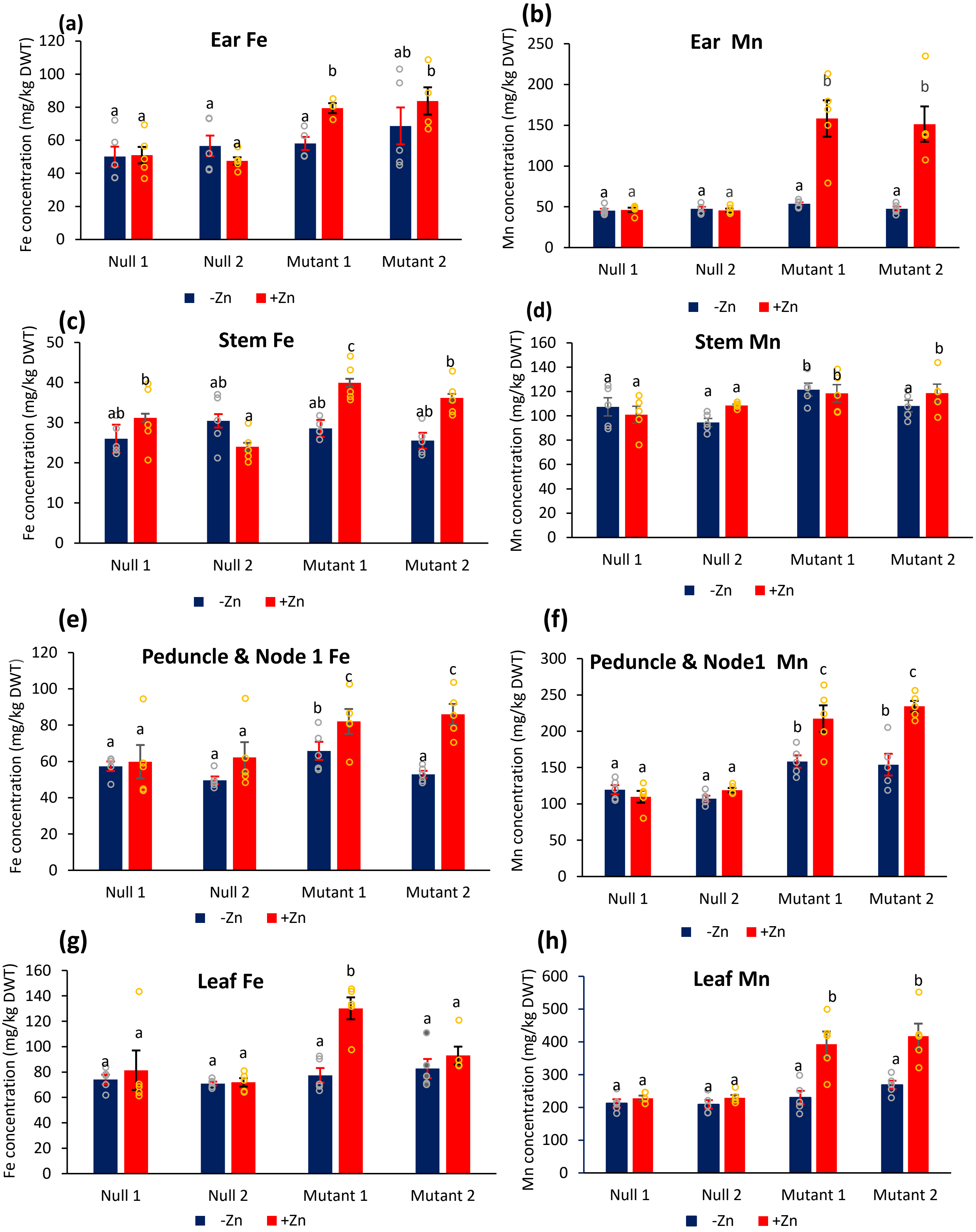

Figure S5. Minerals in different tissues of nulls and triple mutants at anthesis.
The plants were grown in compost without $ZnSO_4$.7H2O (-Zn) and with 200mg $ZnSO_4.7H_2O$ (+Zn) application per pot, and minerals were measured using ICP-OES. (a) Fe concentration in ear. (b) Mn concentration in ear. (c) Fe concentration in stem. (d) Mn concentration in stem. (e) Fe concentration in peduncle and node1. (f) Mn concentration in peduncle and node1. (g) Fe concentration in leaf. (h) Mn concentration in leaf. Data were analysed using two-way multi-stratum ANOVA (n=5) followed by an LSD test for mean comparisons ($P < 0.05$). Error bars represent ± 1 x SE.

(a) **(b)**

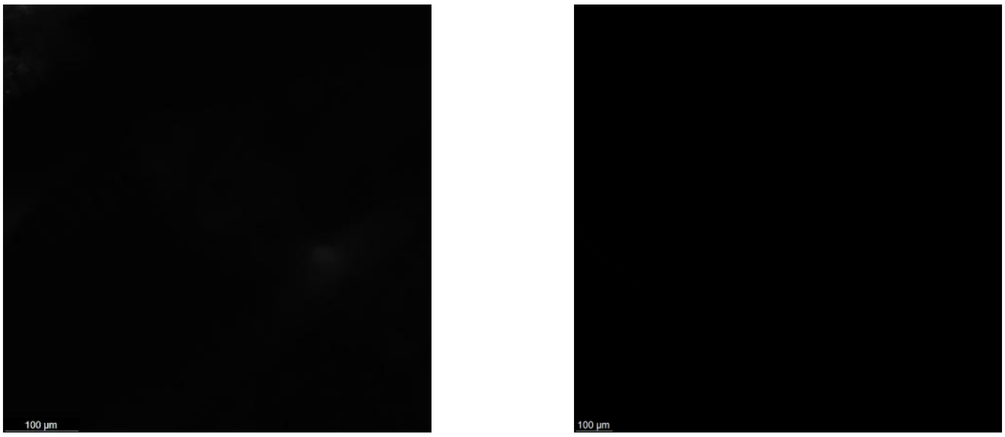

anther node

Figure S6. Negative control of anther and node for Zn localisation using Zinpyr-1.
The slide sections were incubated with 10mM PBS buffer instead of for Zinpyr-1 fluorogenic Zn2+ reporter using confocal microscope. (a) Negative control for anther. (b) Negative control for node 1. scale bars represent 100μm.

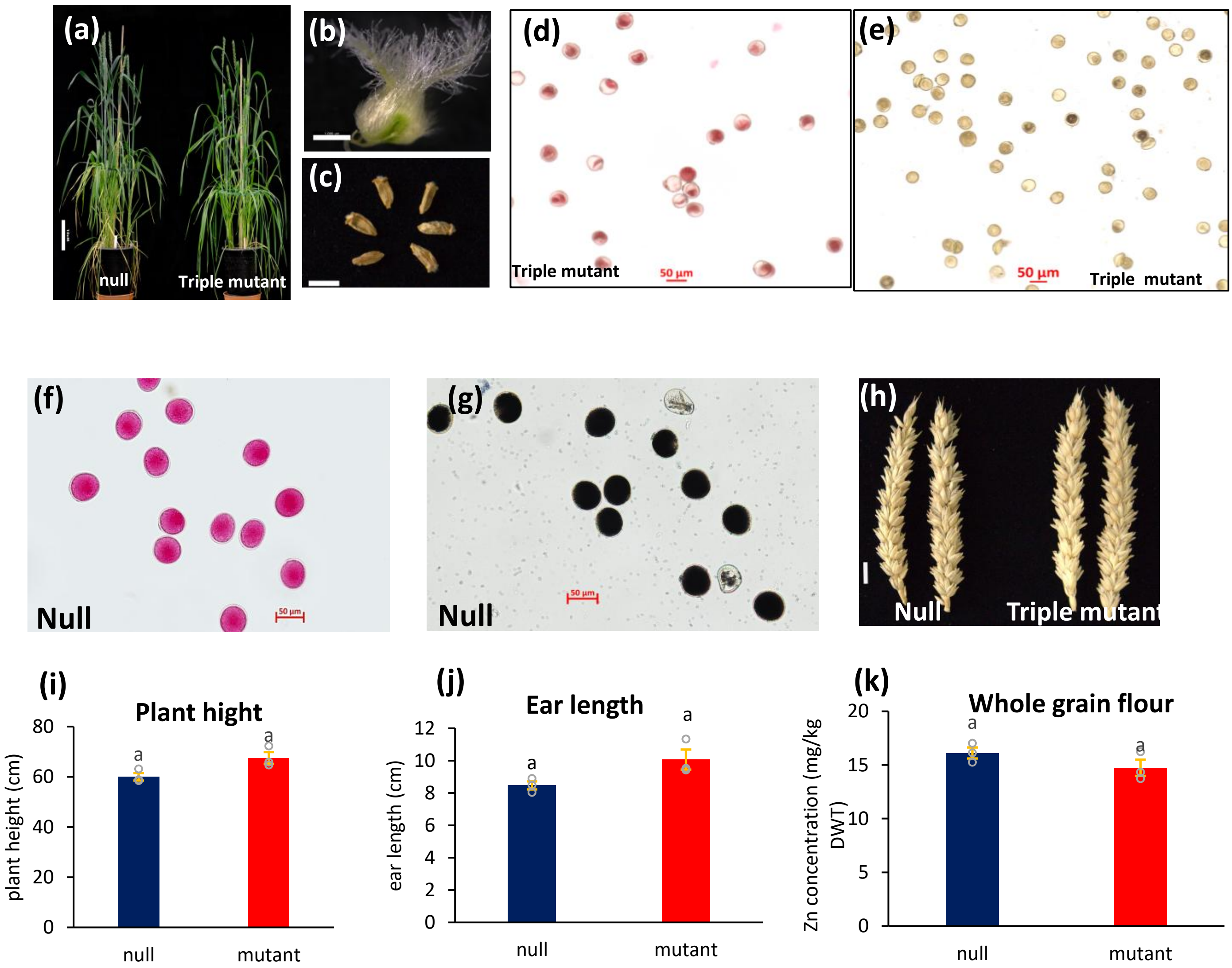

Figure S7. Triple mutants restored their fertility when grown under low or extremely low Zn conditions. (a-e) Triple mutants restored their partial female fertility when they were grown in low fertility sandy soil with minimal organic content containing 20 mg Zn /kg dry weight, half of Zn concentration in compost (40 mg/kg dry weight). (a) Plant height of null and triple mutant at heading stage. (b) Normal carpel morphology of triple mutant. (c) Partial filled seeds from cross of triple mutant ovules (female parent) pollinated with wild type (Cadenza) pollen. (d) Sterile pollen with partial cytoplasm content in triple mutant using Alexander staining. (e) Sterile pollen with minimum starch content using I_2-KI_2 starch staining in triple mutant. (f-k) Triple mutants completely restored their fertility when grown in 50% sand+50% perlite mixture (by volume) supplemented with 0.005µm $ZnSO_4$ in nutrient solution. (f) Viable pollen of nulls were stained using Alexander solution for cytoplasm. (g) Viable pollen of nulls were stained using I_2-KI for starch accumulation. (h) Ear morphology of null and triple mutant. (i) Plant height of nulls and triple mutants. (j) Ear length. (k) Zn concentration in whole grain flour. Data were analysed using exact 19-permutation t-tests with no difference between nulls and triple mutants (n=3). Erro bars represent 1±SE. Scale bars represent 10cm in (a), 1000 µm in (b), 5mm in (c), 50µm in (d-g), 1cm in (h).

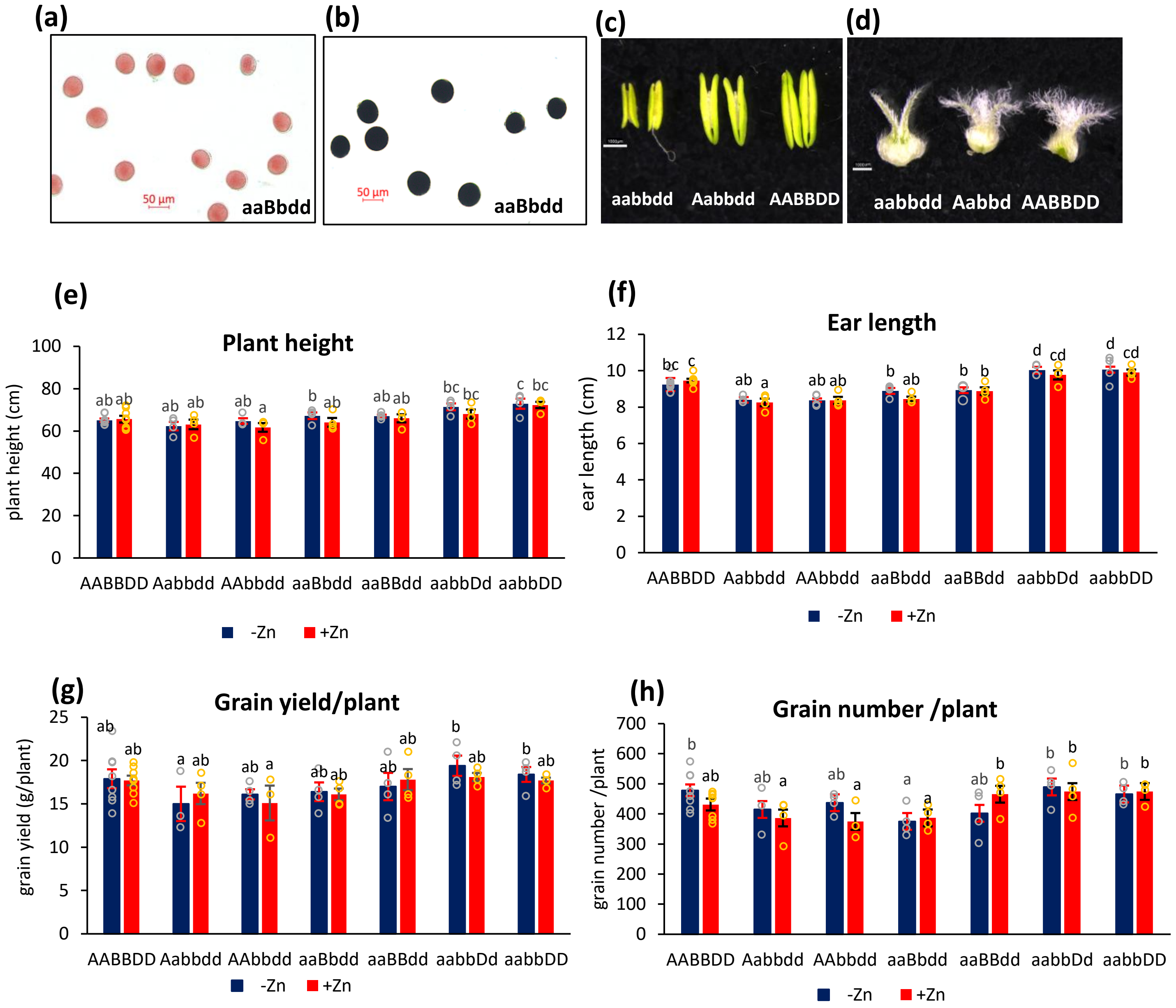

Figure S8. Phenotypes and agronomic traits of partial mutants grown in compost without $ZnSO_4.7H_2O$ (-Zn) and with 200mg $ZnSO_4.7H_2O$ application.

(a) Viable mature pollen in mutant (aaBbdd) using Alexander's staining. (b) Viable mature pollen in mutant (aaBbdd) using I_2-KI staining for starch accumulation. (c) Anther phenotypes of partial mutant (Aabbdd), triple mutant (aabbdd), and null (AABBDD). (d) Carpel phenotype of partial mutant (Aabbdd), triple mutant (aabbdd), and null (AABBDD). (e) Plant height. (f) Ear length. (g) Grain yield per plant. (h) Grain number per plant. Data were analysed using two-way multi-stratum ANOVA with two combined nulls (n=8) and partial mutants (n=4) followed by an LSD test for mean comparisons ($P < 0.05$). Error bars represent ± 1 x SE. Scale bars represent 50µm in (a) and (b), 1000µm in (c) and (d).

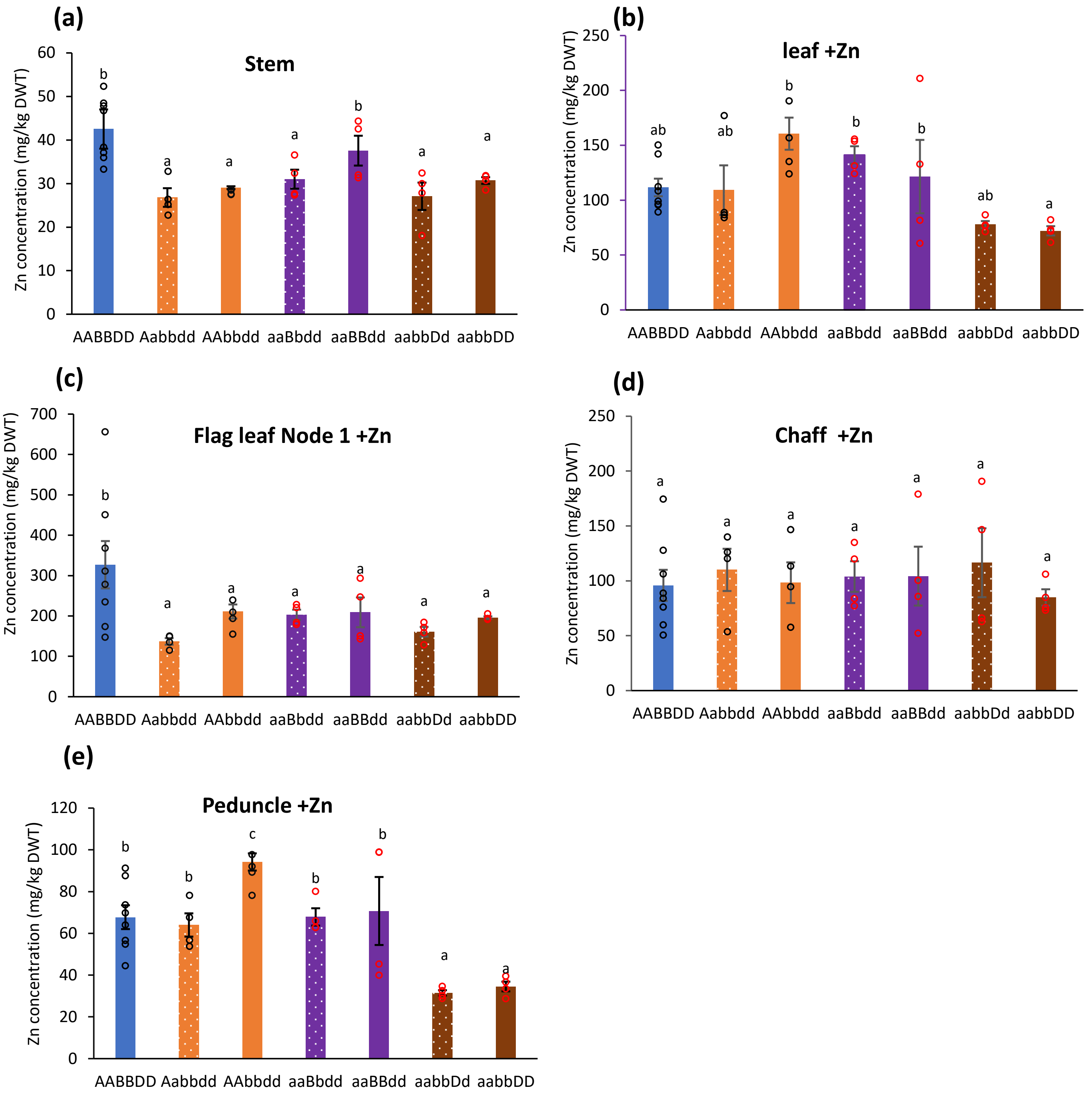

Figure S9. Zn concentrations at mature stage in partial mutant plant tissues grown in compost with 200 mg $ZnSO_4.7H_2O$ application.

Zn concentrations at mature stage were measured using ICP-OES in different tissues of (a) stem, (b) leaf, (c) flag leaf node 1, (d) chaff, and (e) peduncle. Data were analysed using one-way multi-stratum ANOVA with two combined nulls (n=8) and partial mutants (n=4) followed by an LSD test for mean comparisons ($P < 0.05$). Error bars represent ± 1 x SE.

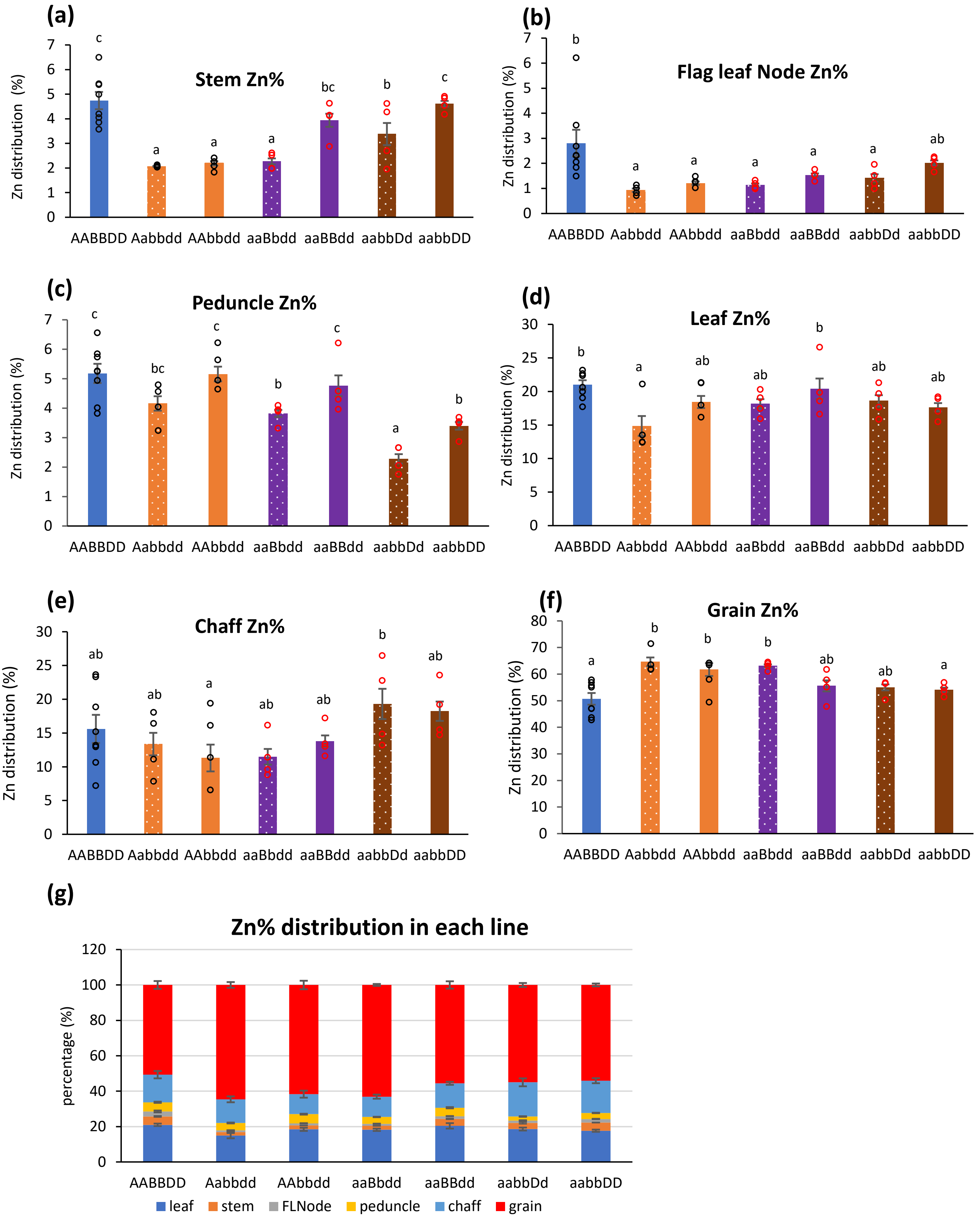

Figure S10. Zn distributions at mature stage in different tissues of partial mutant plants grown in compost with 200mg $ZnSO_4.7H_2O$ (+Zn).
Zn distributions in (a) stem, (b) flag leaf node, (c) peduncle, (d) leaf, (e) chaff, and (f) grain. (g) Zn distributions in each line are shown. FLNode: flag leaf node. Data were analysed using one-way multi-stratum ANOVA with two combined nulls (n=8) and partial mutants (n=4) followed by an LSD test for mean comparisons ($P < 0.05$). Error bars represent ± 1 x SE.

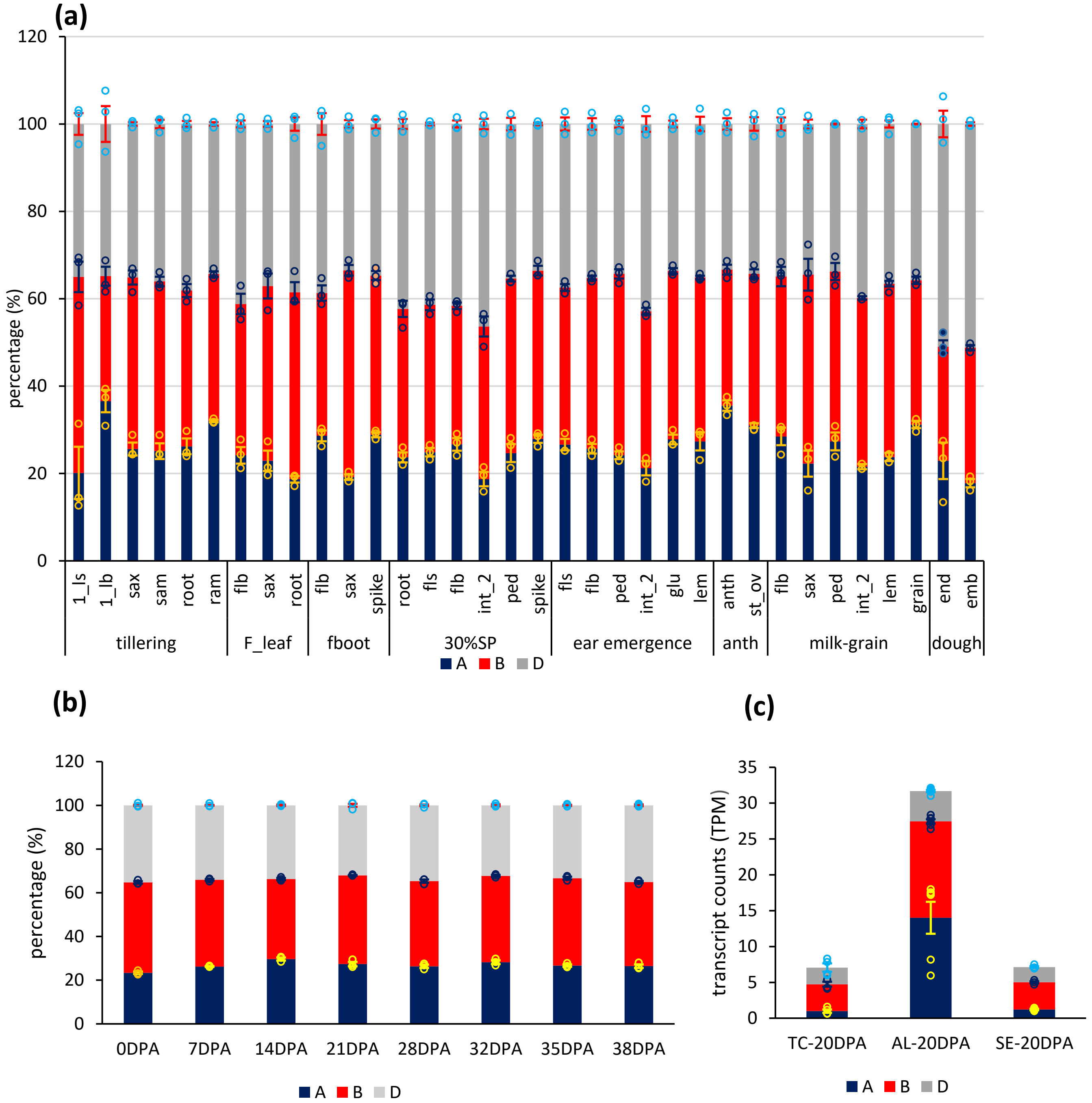

Figure S11. The contribution of homeologs in gene expression during different growth stages. (a)*TaMTP1A, TaMTP1B, and TaMTP1D* percentage of total *TaMTP1* expression in different tissues and growth stages from public RNA-seq data (n=3) of variety (Azhurnaya). Error bars represent ± 1 x SE. 1_ls (1st leaf sheath), 1_lb (1st leaf blade), sax (shoot axis), sam (shoot apical meristem), ram (root apical meristem), flb (flag leaf blade), fls (flag leaf sheath), int_2 (internode below the flag leaf node), ped (peduncle), anth (anther), st_ov (stigma and ovary), glu (glumes), lem (lemma), end (endosperm), emb (embryo); tillering (tillering stage), F-leaf (flag leaf stage), fboot (full booting stage), 30% SP (30% spike out of sheath), anth (anthesis stage), milk-grain (milk grain filling stage), dough (dough grain filling stage). A: *TaMTP1A*, B: *TaMTP1B*, D: *TaMTP1D*. (b) *TaMTP1A, TaMTP1B, and TaMTP1D* percentage of total *TaMTP1* expression in flag leaf node1 from RNA-seq data (n=3) of variety (Paragon) at anthesis (0 days post anthesis, DPA), 7, 14, 21, 28, 32, 35, and 38 DPA. (c) *TaMTP1* expression in grain different cells of Chinese Spring at 20 days post anthesis (DPA) from public RNA-seq data (n=4-6) (Pfeifer K et al, 2014). TC (transfer cell), AL (aleurone cell), SE (starch endosperm), TPM (transcripts per million).
